# Repeatability of Cardiac Magnetic Resonance Radiomics: A Multi-Centre Multi-Vendor Test-Retest Study

**DOI:** 10.3389/fcvm.2020.586236

**Published:** 2020-12-02

**Authors:** Zahra Raisi-Estabragh, Polyxeni Gkontra, Akshay Jaggi, Jackie Cooper, João Augusto, Anish N. Bhuva, Rhodri H. Davies, Charlotte H. Manisty, James C. Moon, Patricia B. Munroe, Nicholas C. Harvey, Karim Lekadir, Steffen E. Petersen

**Affiliations:** ^1^NIHR Barts Biomedical Research Centre, William Harvey Research Institute, Queen Mary University of London, London, United Kingdom; ^2^Barts Heart Centre, St Bartholomew's Hospital, Barts Health NHS Trust, London, United Kingdom; ^3^Departament de Matemàtiques i Informàtica, Universitat de Barcelona, Barcelona, Spain; ^4^Institute of Cardiovascular Science, University College London, London, United Kingdom; ^5^MRC Lifecourse Epidemiology Unit, University of Southampton, Southampton, United Kingdom; ^6^NIHR Southampton Biomedical Research Centre, University of Southampton and University Hospital Southampton NHS Foundation Trust, Southampton, United Kingdom

**Keywords:** radiomics, test-retest, repeatability, reproducibility, cardiovascular magnetic resonance, texture analysis

## Abstract

**Aims:** To evaluate the repeatability of cardiac magnetic resonance (CMR) radiomics features on test-retest scanning using a multi-centre multi-vendor dataset with a varied case-mix.

**Methods and Results:** The sample included 54 test-retest studies from the VOLUMES resource (thevolumesresource.com). Images were segmented according to a pre-defined protocol to select three regions of interest (ROI) in end-diastole and end-systole: right ventricle, left ventricle (LV), and LV myocardium. We extracted radiomics shape features from all three ROIs and, additionally, first-order and texture features from the LV myocardium. Overall, 280 features were derived per study. For each feature, we calculated intra-class correlation coefficient (ICC), within-subject coefficient of variation, and mean relative difference. We ranked robustness of features according to mean ICC stratified by feature category, ROI, and cardiac phase, demonstrating a wide range of repeatability. There were features with good and excellent repeatability (ICC ≥ 0.75) within all feature categories and ROIs. A high proportion of first-order and texture features had excellent repeatability (ICC ≥ 0.90), however, these categories also contained features with the poorest repeatability (ICC < 0.50).

**Conclusion:** CMR radiomic features have a wide range of repeatability. This paper is intended as a reference for future researchers to guide selection of the most robust features for clinical CMR radiomics models. Further work in larger and richer datasets is needed to further define the technical performance and clinical utility of CMR radiomics.

**Graphical Abstract:**
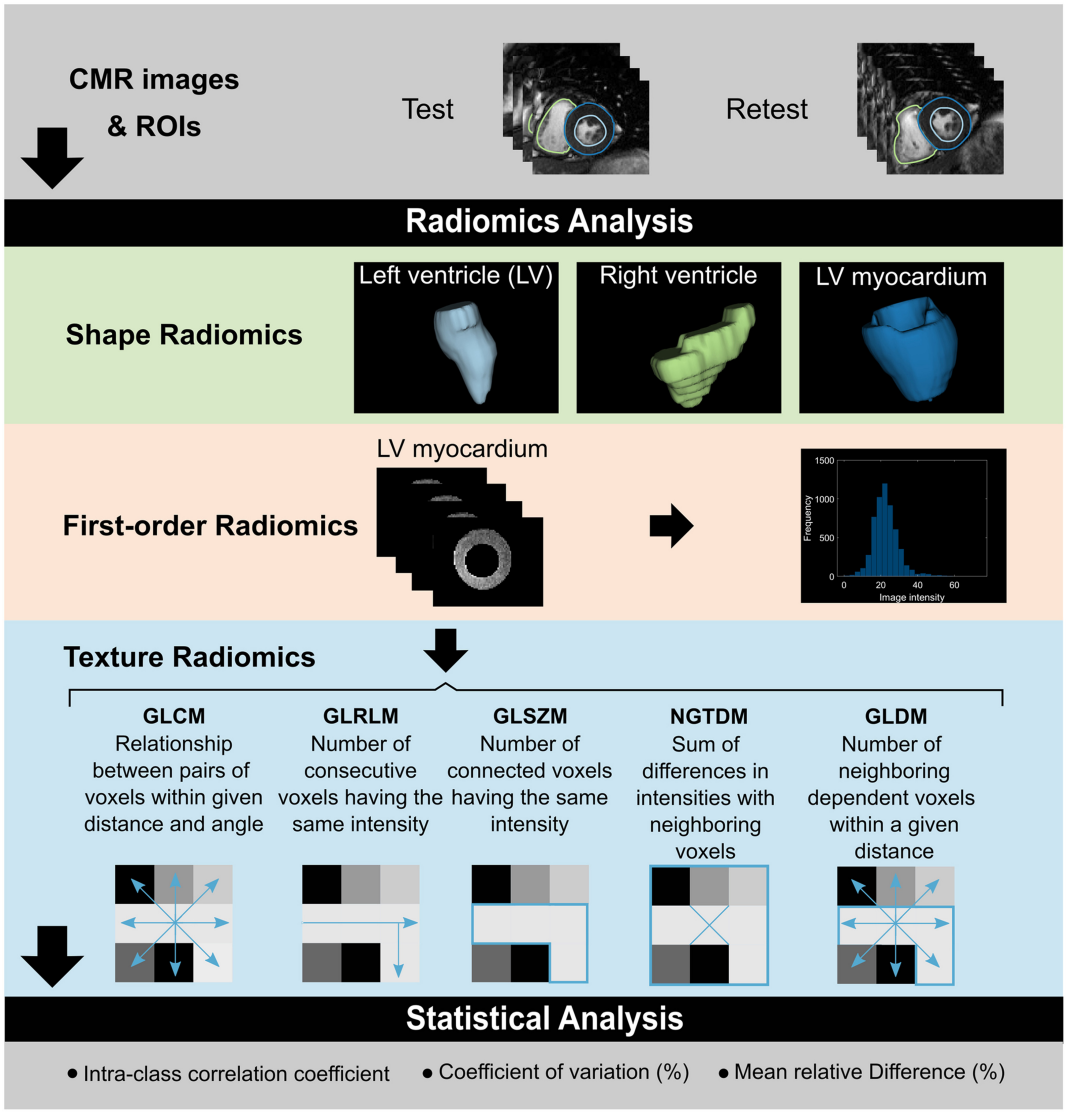
Overview of the pipeline to evaluate test-retest repeatability of CMR radiomics features. Test-retest CMR studies are segmented to define three ROIs for radiomics analysis: LV blood pool, RV blood pool, and LV myocardium. Shape features are analyzed for all three ROIs. Additionally, first-order and texture features are extracted from the LV myocardium. Statistical analysis is performed to assess repeatability performance of radiomics features. CMR, cardiac magnetic resonance; GLCM, gray level co-occurrence matrix; GLDM, gray level dependence matrix; GLRLM, gray level run length matrix; GLSZM, gray level size zone matrix; NGTDM, neighboring gray tone difference matrix; ROI, region of interest.

## Introduction

Radiomics is an image analysis technique whereby a large number of advanced quantitative features are extracted from voxel level data of routine-care medical images (1). Radiomics data are structured in a minable format and can be used to develop models which link image features with biological phenotypes. The over-arching aim of radiomics analysis is to develop models for faster and more accurate disease diagnosis and risk prediction.

Radiomics features comprise (1) shape and (2) signal intensity-based features (Graphical abstract). Shape features include geometric quantifiers of the rendered volume, such as total volume, surface area, and descriptors of overall shape, such as sphericity, elongation, and compactness. Intensity-based radiomics features describe the global distribution (first-order features) and pattern (texture features) of voxel signal intensities. First-order features describe the distribution of signal intensities of individual voxels, without consideration to spatial relationships. They are derived from histogram-based methods and summarize the intensity levels in the defined region of interest (ROI) into single quantifiers such as mean, median, maximum, randomness (entropy), skewness (asymmetry), and kurtosis (flatness). Texture features are statistical descriptors of the relationships between neighboring voxels of similar (or different) signal intensities. They are calculated using various matrix analysis methods according to standardized mathematical definitions.

The clinical utility of radiomics models for diagnosis, surveillance, and prognostication has been repeatedly demonstrated within the context of oncology (2–7). Application of radiomics analysis to cardiac magnetic resonance (CMR) images is in its early developmental stages (1). Proof-of-concept studies have demonstrated incremental value of CMR radiomics models in distinguishing important disease entities such as hypertensive heart disease and hypertrophic cardiomyopathy (8), identification of myocardial infarction from non-contrast images (9–11), and prediction of life-threatening arrhythmias (12). Thus, CMR radiomics features may have potential as important novel quantitative imaging biomarkers (QIBs).

Translation of CMR radiomics to clinical practice requires external validity of proposed models. A key determinant of model performance in clinical and pre-clinical settings is repeatability, that is, the ability to repeatedly measure the same feature under identical or near-identical conditions on the same measurement unit (subject/phantom). CMR radiomics features are subject to technical (image acquisition, artifact, image processing) and population-related variations. However, their repeatability performance has not been adequately assessed in existing work. Such analysis is an essential step in assessing the clinical utility of this methodology, both for the underpinning research and the eventual clinical implementation.

We present, to the best of our knowledge, the first evaluation of the repeatability of CMR radiomics features on test-retest scanning using a multi-centre multi-vendor dataset with a varied case-mix. This paper is intended as a reference for future researchers to guide selection of the most robust features for inclusion in CMR radiomics models.

The design, terminology, and statistical methods reflect recommendations from the Quantitative Imaging Biomarker Alliance (QIBA) (13, 14). QIBA is a group of the Radiological Society of North America established to guide standardization of the development and validation of QIBs. Reporting of methods is in line with relevant aspects of the Radiomics Quality Score (RQS) (15). The RQS provides guidance to improve quality and transparency of reporting in radiomics studies.

## Methods

### Setting and Study Population

We analyzed a subset of studies from the VOLUMES resource (16), comprising test-retest studies from five centres across the United Kingdom (Barts Heart Centre, University Hospitals Bristol, Leeds Teaching Hospitals, University College London Hospital, University Hospitals Birmingham NHS Trusts). The sample included a varied mix of disease and healthy cases. Exclusion criteria included age < 18 years-old, implantable cardiac devices, significant arrhythmia, claustrophobia, and poor breath-holding. Further information about the resource, acquisition protocols, and study population are detailed in a dedicated publication and online resource (16, 17).

### Scanning Protocol

Two vendors (Philips, Siemens), three models (Achieva, Avanto, Aera), and two magnet strengths (1.5 Tesla, 3 Tesla) were used. Scanning protocols across all contributing centres were in accordance with international recommendations (18). Complete short axis stacks covering the left and right ventricles (LV, RV) were acquired using balanced steady state free precession sequences. Details of acquisition parameters are summarized in Supplementary Table 1. Test-retest studies were performed under repeatability conditions with the same patient, location, scanner, acquisition protocol, and operating conditions. The time interval between test and retest was between 0 and 7 days. Given this very short test-retest interval, it is highly unlikely that any change in radiomics features could be due to alterations in the underlying cardiovascular health. Individuals having both scans on the same day were repositioned prior to retest with repeat isocentre positioning.

### Image Segmentation

Image segmentation was performed blind to details of image acquisition, patient information, diagnosis, or scan pairings. LV endocardial and epicardial and RV endocardial contours were drawn in end-diastole and end-systole on short-axis stack images to select three ROIs for radiomics analysis: RV blood pool, LV blood pool, and LV myocardium. The blood pool ROIs reflect LV and RV cavities in end-diastole and end-systole. Segmentation was performed according to a pre-defined standard operating procedure (SOP) (19). Papillary muscles were considered part of the LV blood pool; the basal LV slice was included if there was >50% myocardium circumferentially, and for the RV, volumes below the pulmonary valve were included with position judged by review of cine images and orthogonal cuts. Contours were drawn using a machine learning approach with expert edits using Circle® cardiovascular imaging version 5.11.0 (Circle cardiovascular imaging Inc., Calgary, Canada). Initial checks and adjustments were made by Z.R.E., trainee cardiologist with 2-years' experience in CMR and dedicated training in the SOP, and cross-checked by S.E.P., consultant cardiologist with over 15-years' experience with CMR.

### Radiomics Feature Extraction

**Figure 1:**
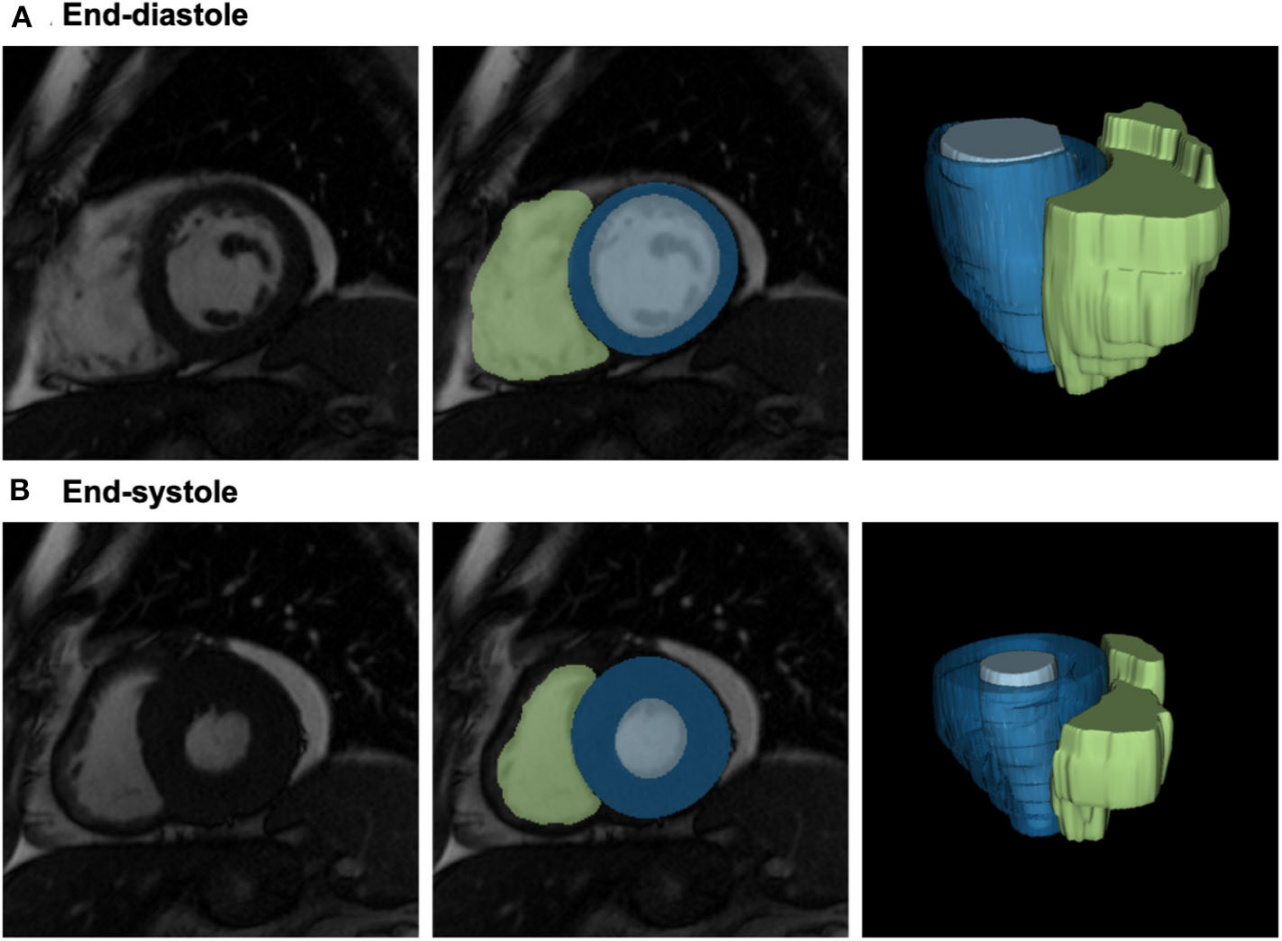
Definition of the LV/RV blood pool and the LV myocardium for radiomics analysis. From left to right: 2D short axis mid-ventricular slice; segmentation of the three regions of interest shown overlaid on the image: LV myocardium (blue), LV blood pool (light blue), and RV blood pool (green); 3D reconstructions of the segmented ROIs. Please note, that radiomics analysis has been performed in 3D; 2D slices are provided for visualization purposes only. CMR: cardiac magnetic resonance; LV: left ventricle; ROI: region of interest; RV: right ventricle.

Radiomics feature extraction was performed blind to details of image acquisition, patient information, diagnosis, or scan pairings. Contours from the image segmentation were used to create 3D image masks for the three ROIs in end-diastole and end-systole (Figure 1). Toward this, voxels belonging to the three ROIs were indicated as foreground voxels using a unique label per ROI, whilst all other voxels were defined as background. An in-house software implemented in Python was used to convert the contours into binary masks. In brief, the image contour was parsed into an xml file that contains the coordinates of all contour points. Subsequently, a polygon was built joining the points in the coordinate space to form the mask. Lastly, the area bounded by the contour in every slice is filled with ones using OpenCV function, fillpoly, resulting in the binary ROI. The process was repeated for all delineated contours. The image masks and the corresponding CMR DICOM® (Digital Imaging and Communications in Medicine) images were converted to NIFTI (Neuroimaging Informative Technology Initiative) format for subsequent processing.

Radiomics features were extracted from the 3D CMR images and the corresponding 3D mask (i.e., the full 3D CMR and mask volumes) using the open-source python-based PyRadiomics platform version 2.2.0 in end-diastole and end-systole. No pre-processing or re-segmentation was used before computing the features. We considered all features available in Pyradiomics including older versions in an effort to provide robustness insights for features, that although currently considered deprecated, were largely used in the past.

Overall, 16 shape, 19 first-order, and 73 texture features were available, we applied all feature categories to the LV myocardium, and shape features to the LV and RV blood pool ROIs. For gray value discretisation, we used a fixed bin width of 25 intensity values. The texture features were extracted using five different matrices: gray-level co-occurrence matrix (GLCM, 23 features), gray-level run-length matrix (GLRLM, 16 features), gray-level size-zone matrix (GLSZM, 15 features), neighboring gray tone difference matrix (NGTDM, 5 features), and gray-level dependence matrix (GLDM, 14 features). In total, 280 features across the three ROIs, two phases, and three radiomics categories (shape, first-order, texture) were calculated per study.

### Statistical Analysis

We considered intra-class correlation coefficient (ICC) as a valid aggregate summary of repeatability performance in this setting. For calculation of ICC, we used a one-way random effects model for absolute agreement based on a single measure; as the two time points (test, retest) can be considered interchangeable, the one-way model is valid and appropriate for our analysis (20). For each radiomics feature, we calculated the ICC and corresponding 95% confidence interval using the variance components from a one-way ANOVA (analysis of variance). We assigned descriptive terms to ICC values in line with published guidance on ICC interpretation (20): <0.5 poor, 0.5–0.75 moderate, 0.75–0.9 good, ≥0.9 excellent. We ranked robustness of features according to the mean ICC stratified by feature category, ROI, and cardiac phase. In addition, for each feature, we report within-subject variability expressed through within-subject coefficient of variation (CV) and mean relative difference. We present Bland-Altman plots for a selection of exemplar features from different levels of repeatability.

## Results

### Population Characteristics

The sample included 54 paired test-retest CMR scans of 40 men and 14 women with mean (standard deviation) age of 51.9 (±16.8) years. Nine subjects were healthy volunteers. The remainder had a range of ischaemic and non-ischaemic cardiovascular conditions (Table 1). The majority of scans were performed on 1.5 Tesla Siemens scanners (Aera, Avanto). Three cases were performed on 3 Tesla Philips Achieva scanners. The interval between test and retest was no more than 7 days and for the majority, both scans were performed on the same day (85%, *n* = 46).

**Table 1:** Characteristic of the study population.

**DEMOGRAPHICS**	
Age (mean ±standard deviation)	51.9 (±16.8) years
Sex (Men: *n*, percentage)	40 (74%)
**DIAGNOSIS (*****n*****)**	
Healthy volunteer	9
Myocardial infarction (chronic)	14
Dilated cardiomyopathy	5
Hypertrophic cardiomyopathy	15
Left ventricular hypertrophy	4
Cardio-oncology	7
**SCANNER VENDOR, MODEL, MAGNET STRENGTH (*****n*****)**	
Siemens, Aera, 1.5 Tesla	23
Siemens Avanto, 1.5 Tesla	28
Philips Achieva, 3 Tesla	3

### Repeatability of Conventional CMR Indices

We first studied the repeatability of conventional CMR indices to assess possible loss of robustness associated with the segmentation process. We calculated ICC, CV, and mean relative difference for LV end-diastolic volume, LV end-systolic volume, LV ejection fraction, LV mass, RV end-diastolic volume, RV end-systolic volume, and RV ejection fraction (Supplementary Table 1). There was excellent repeatability for LV end-diastolic volume (ICC 0.97, 95% CI 0.96–0.99), LV end-systolic volume (ICC 0.96, 95% CI 0.93–0.98), and LV mass (ICC 0.95, 95% CI 0.91–0.97). As expected, repeatability of the RV indices, was slightly lower than that of the LV. Thus, we confirmed good quality contouring with repeatability of conventional CMR indices overall exceeding that of previous reports (19).

### Repeatability of LV Blood Pool Shape Features

**Figure 2:**
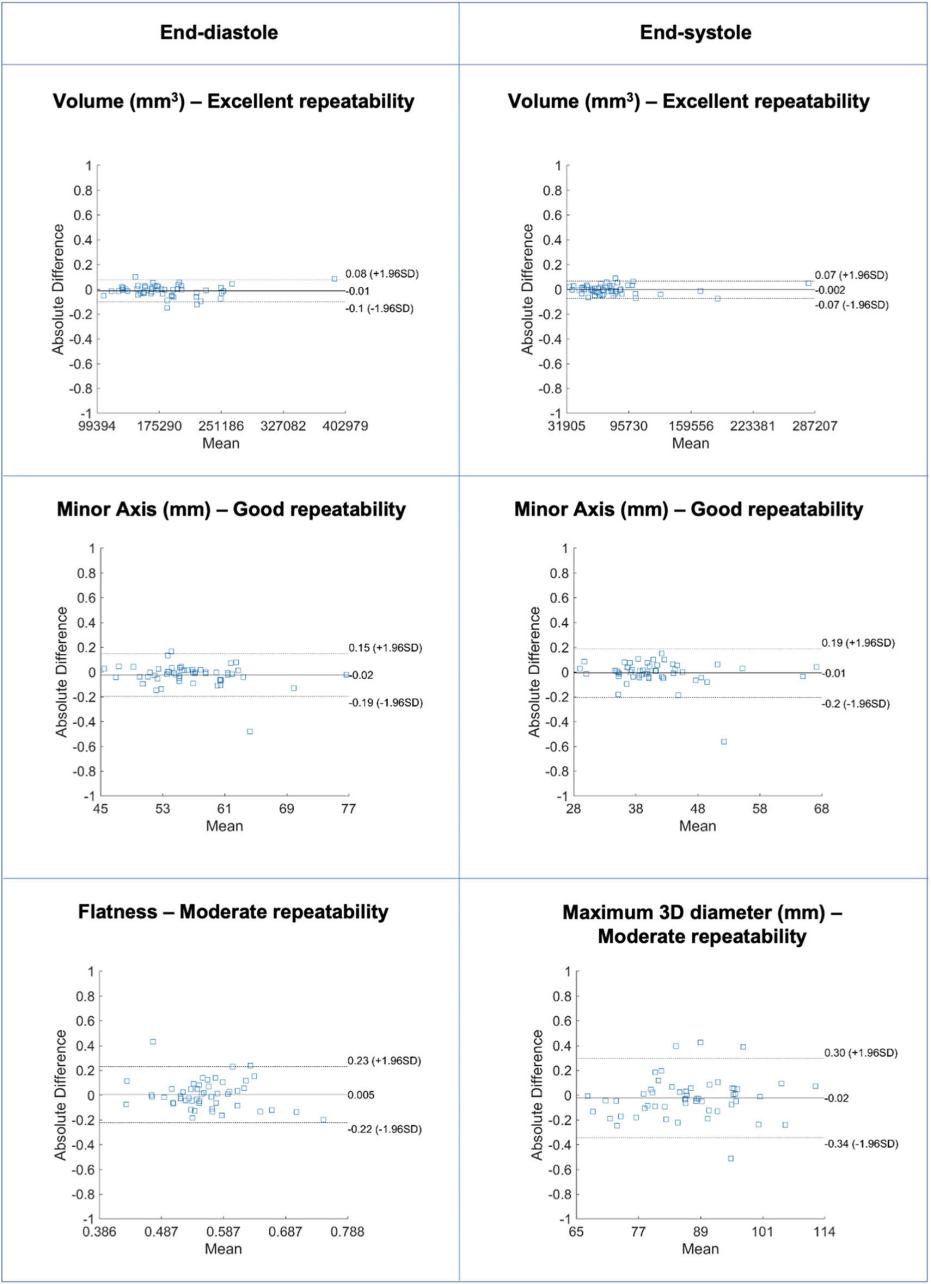
Bland-Altman plots for selected LV blood pool shape features in end-diastole (left) and end-systole (right) with different levels of repeatability. Differences in Bland-Altman are calculated after normalizing radiomics in the range [0–1] to facilitate comparison among different features. All features are unitless. LV: left ventricle.

**Figure 3:**
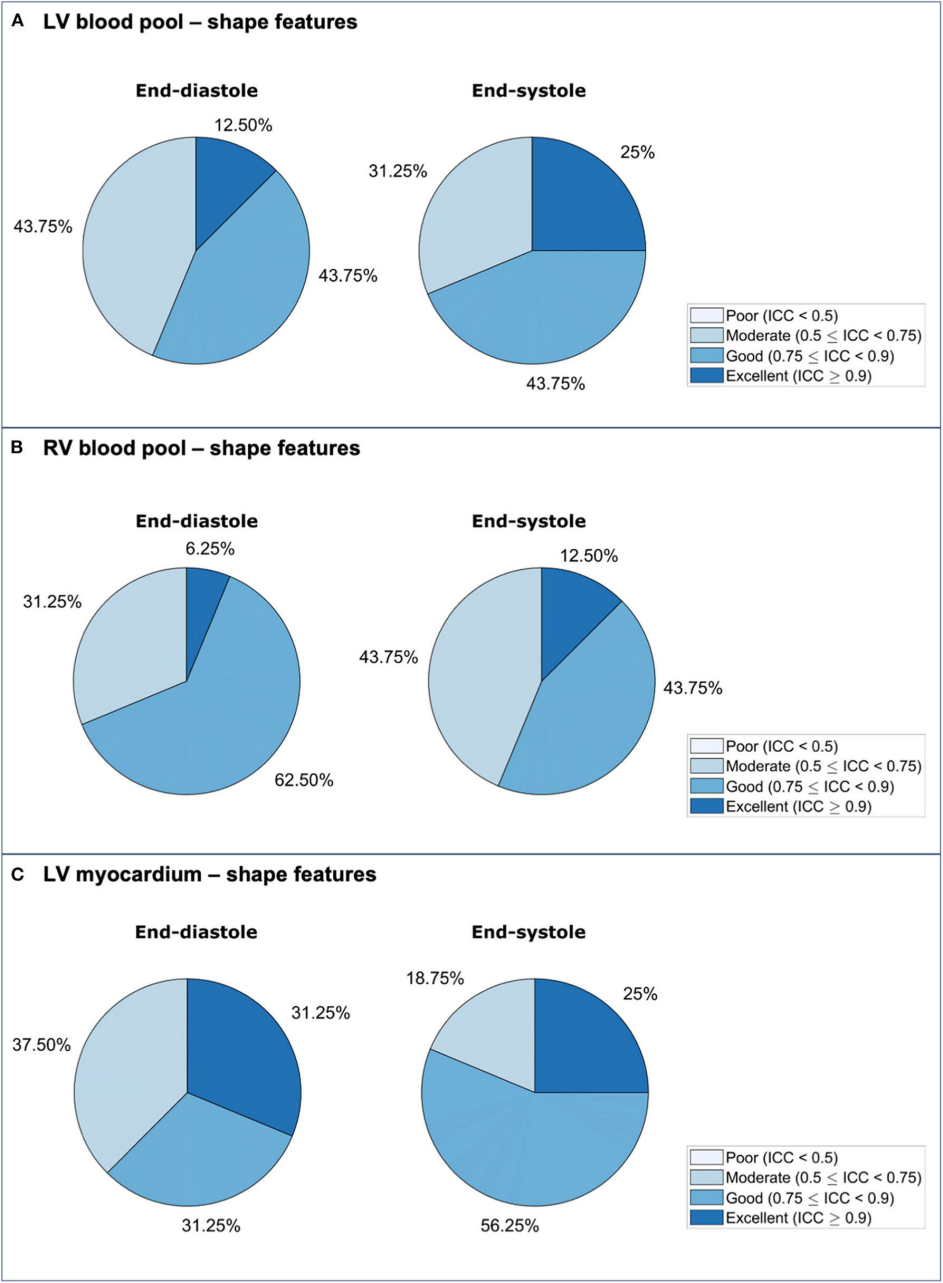
Repeatability of radiomics shape features for the LV blood pool **(A)**, RV blood pool **(B)**, and LV myocardium **(C)** in end-diastole and end-systole. ICC: intra-class correlation coefficient; LV: left ventricle; RV: right ventricle.

Repeatability of LV blood pool shape features varied from moderate to excellent with mean ICC ranging from 0.511 to 0.974 [Median (IQR): 0.871 (0.175)] (Table 2, Supplementary Table 2, Figure 2). Overall, there was better repeatability in end-systole than in end-diastole (Figure 3A). The most robust features were “volume” in both end-systole and end-diastole, “least axis length” in end-diastole, and “surface area” in end-systole. In both end-diastole and end-systole, the least robust features were “spherical disproportion,” “sphericity,” “compactness,” and “compactness 2.”

**Table 2:** Repeatability of left ventricular blood pool shape features in end-diastole.

**Feature name**	**Robustness**	**ICC (95% CI)**	**CV (%)**	**MRD (%)**
Volume	Excellent	0.957 (0.927, 0.975)	5.35	5.58
Least axis length	Excellent	0.950 (0.916, 0.971)	2.39	2.51
Minor axis length	Good	0.879 (0.800, 0.928)	3.35	2.93
Surface area	Good	0.876 (0.796, 0.926)	5.77	5.75
Surface area to volume ratio	Good	0.869 (0.785, 0.921)	3.46	3.5
Maximum 2D diameter (slice)	Good	0.844 (0.747, 0.906)	4.15	4.29
Maximum 2D diameter (column)	Good	0.777 (0.646, 0.864)	4.34	4.96
Elongation	Good	0.775 (0.642, 0.863)	5.7	5.94
Major axis length	Good	0.764 (0.626, 0.856)	4.72	4.75
Flatness	Moderate	0.747 (0.602, 0.845)	5.9	6.06
Maximum 2D diameter (row)	Moderate	0.746 (0.601, 0.844)	4.95	5.3
Maximum 3D diameter	Moderate	0.698 (0.532, 0.813)	5.19	5.64
Compactness 2	Moderate	0.575 (0.367, 0.729)	10.55	9.39
Compactness	Moderate	0.554 (0.339, 0.714)	5.34	4.72
Sphericity	Moderate	0.546 (0.329, 0.708)	3.57	3.15
Spherical disproportion	Moderate	0.511 (0.285, 0.683)	3.57	3.15

*CI, confidence interval; CV, coefficient of variation; ICC, intra-class correlation coefficient; MRD, mean relative difference*.

### Repeatability of RV Blood Pool Shape Features

**Figure 4:**
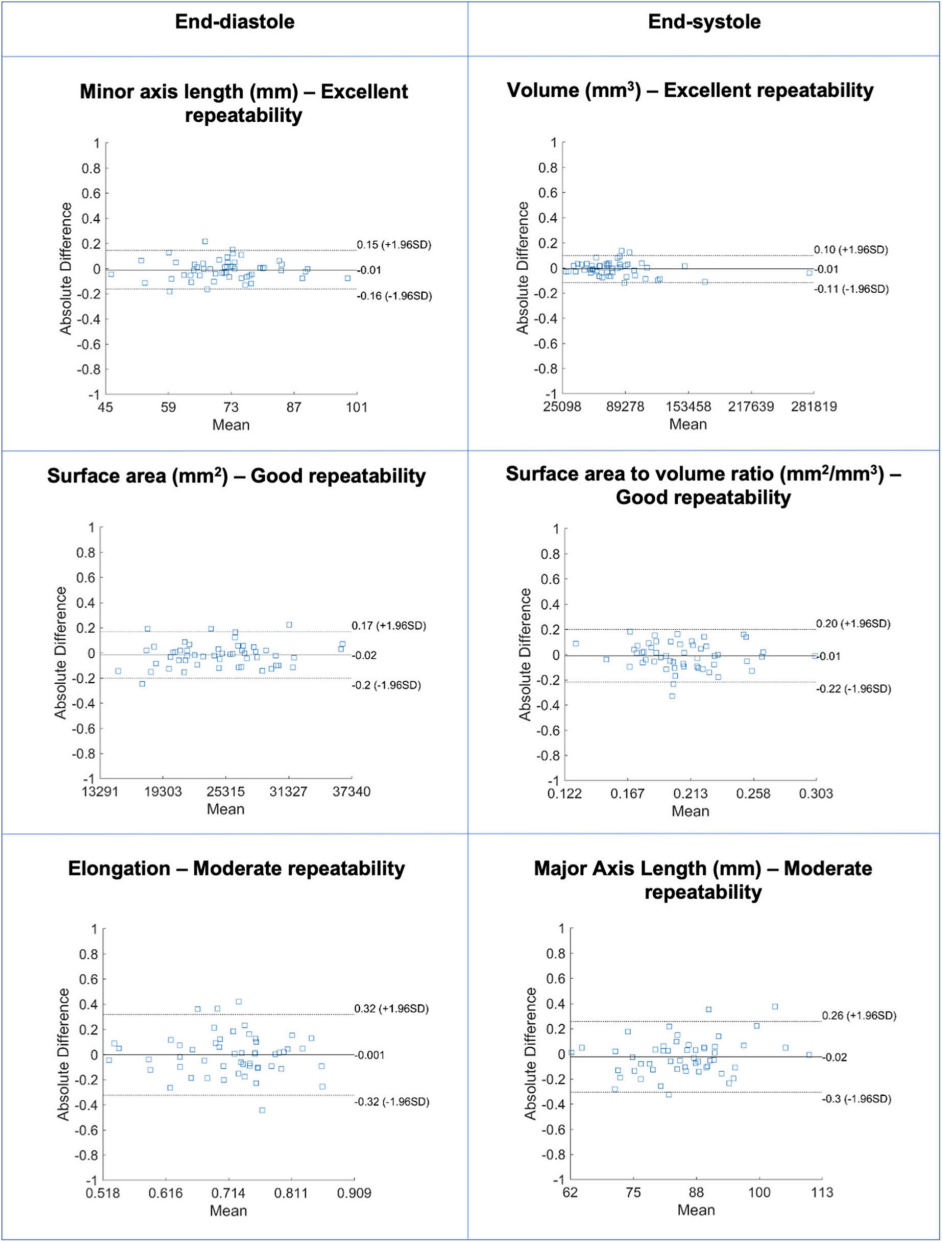
Bland-Altman plots for selected RV blood pool shape features in end-diastole (left) and end-systole (right) with different levels of repeatability. Differences in Bland-Altman are calculated after normalizing radiomics in the range [0–1] to facilitate comparison among different features. All features are unitless. RV: right ventricle.

Repeatability of RV blood pool shape features varied from moderate to excellent with mean ICC ranging from 0.556 to 0.941 [Median (IQR): 0.793 (0.158)] (Table 3, Supplementary Table 3, Figure 4). Overall, there was better repeatability in end-diastole than in end-systole (Figure 3B). The most robust RV shape features were “volume” in end-diastole, “minor axis length” in end-systole, and “surface area” in both phases. As for the LV blood pool, “spherical disproportion,” “sphericity,” “compactness 2,” and “compactness” had the poorest repeatability across both cardiac phases.

**Table 3:** Repeatability of right ventricular blood pool shape features in end-diastole.

**Feature name**	**Robustness**	**ICC (95% CI)**	**CV (%)**	**MRD (%)**
Minor axis length	Excellent	0.915 (0.858, 0.950)	4.52	4.87
Surface area	Good	0.899 (0.832, 0.940)	7.38	7.57
Volume	Good	0.894 (0.825, 0.937)	11.03	11.52
Least axis length	Good	0.841 (0.741, 0.904)	4.34	4.6
Maximum 2D diameter (slice)	Good	0.837 (0.736, 0.902)	4.36	4.26
Surface area to volume ratio	Good	0.816 (0.704, 0.889)	5.45	5.96
Flatness	Good	0.800 (0.679, 0.878)	5.55	6.04
Maximum 3D diameter	Good	0.795 (0.672, 0.876)	5.33	5.69
Major axis length	Good	0.791 (0.666, 0.873)	4.98	5.02
Maximum 2D diameter (row)	Good	0.790 (0.665, 0.873)	5.91	6.5
Maximum 2D diameter (column)	Good	0.772 (0.638, 0.861)	6.8	7.42
Elongation	Moderate	0.749 (0.604, 0.846)	6.22	6.73
Compactness	Moderate	0.679 (0.506, 0.800)	4.78	5.35
Compactness 2	Moderate	0.679 (0.506, 0.800)	9.52	10.67
Sphericity	Moderate	0.679 (0.505, 0.800)	3.19	3.57
Spherical disproportion	Moderate	0.672 (0.496, 0.795)	3.19	3.57

*CI, confidence interval; CV, coefficient of variation; ICC, intra-class correlation coefficient; MRD, mean relative difference*.

### Repeatability of LV Myocardium Shape Features

**Figure 5:**
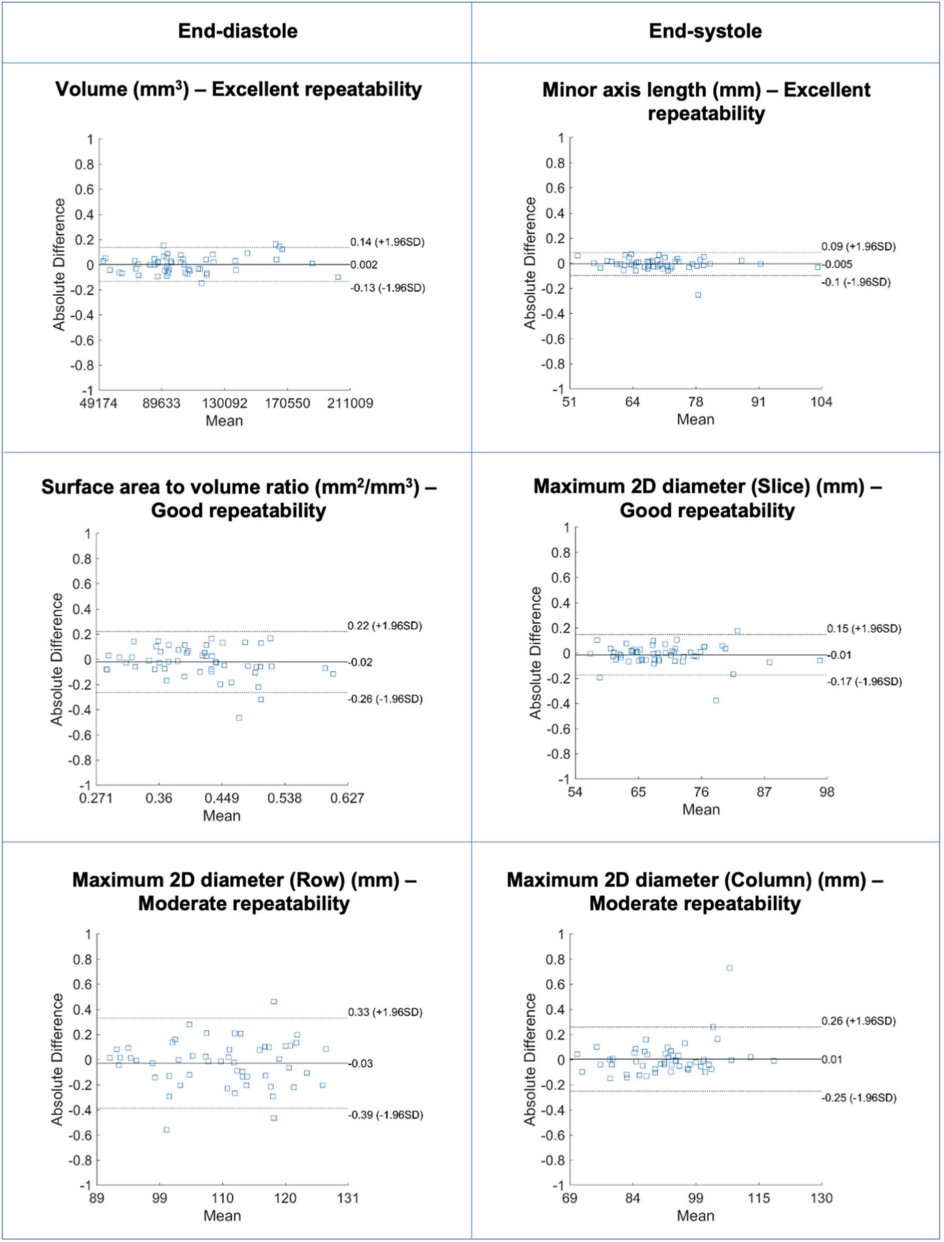
Bland-Altman plots for selected LV myocardium shape features in end-diastole (left) and end-systole (right) with different levels of repeatability. Differences in Bland-Altman are calculated after normalizing radiomics in the range [0–1] to facilitate comparison among different features. All features are unitless. LV: left ventricle.

Repeatability of LV myocardium shape features varied from moderate to excellent with mean ICC ranging from 0.544 and 0.96 [Median (IQR): 0.839 (0.172)] (Table 4, Supplementary Table 4, Figure 5). As with the LV blood pool shape features, there was better repeatability of myocardial shape features in end-systole than in end-diastole (Figure 3C). The most robust features in both end-diastole and end-systole were “minor axis length,” “least axis length,” “surface area,” and “volume.” The least robust features were “flatness” and “maximum 3D diameter” in both cardiac phases.

**Table 4:** Repeatability of left ventricular myocardium shape features in end-diastole.

**Feature name**	**Robustness**	**ICC (95% CI)**	**CV (%)**	**MRD (%)**
Volume	Excellent	0.946 (0.909, 0.968)	7.34	8.6
Minor axis length	Excellent	0.944 (0.905, 0.967)	2.27	2.53
Least axis length	Excellent	0.934 (0.890, 0.961)	2.62	2.7
Maximum 2D diameter (slice)	Excellent	0.913 (0.855, 0.948)	2.88	2.9
Surface area	Excellent	0.909 (0.849, 0.946)	5.23	5.79
Surface area to volume ratio	Good	0.837 (0.735, 0.902)	7.03	7.89
Maximum 2D diameter (column)	Good	0.779 (0.649, 0.866)	4.09	4.76
Compactness 2	Good	0.761 (0.622, 0.854)	15.91	17.81
Compactness	Good	0.757 (0.616, 0.851)	8.06	8.97
Sphericity	Good	0.753 (0.610, 0.848)	5.39	5.99
Maximum 2D diameter (row)	Moderate	0.739 (0.590, 0.839)	4.88	5.23
Spherical disproportion	Moderate	0.724 (0.569, 0.830)	5.39	5.99
Major axis length	Moderate	0.717 (0.559, 0.825)	5.06	5.27
Elongation	Moderate	0.693 (0.525, 0.809)	5.44	5.38
Maximum 3D diameter	Moderate	0.677 (0.503, 0.799)	5.16	5.61
Flatness	Moderate	0.544 (0.327, 0.707)	6.45	6.25

*CI, confidence interval; CV, coefficient of variation; ICC, intra-class correlation coefficient; MRD, mean relative difference*.

### Shape Feature Trends Across Regions of Interest

Across all three regions of interest and the two phases, “volume” and “surface area” followed by measures of the heart short axis, i.e., “least axis length” and “minor axis length,” showed the highest average repeatability (Supplementary Figure 1). The correlated sphericity-measuring features, i.e., “spherical disproportion,” “sphericity,” “compactness 1,” and “compactness 2,” produced the lowest average reproducibility and greatest variance in reproducibility across all regions (Supplementary Figure 1).

### Repeatability of LV Myocardium First-Order Features

**Figure 6:**
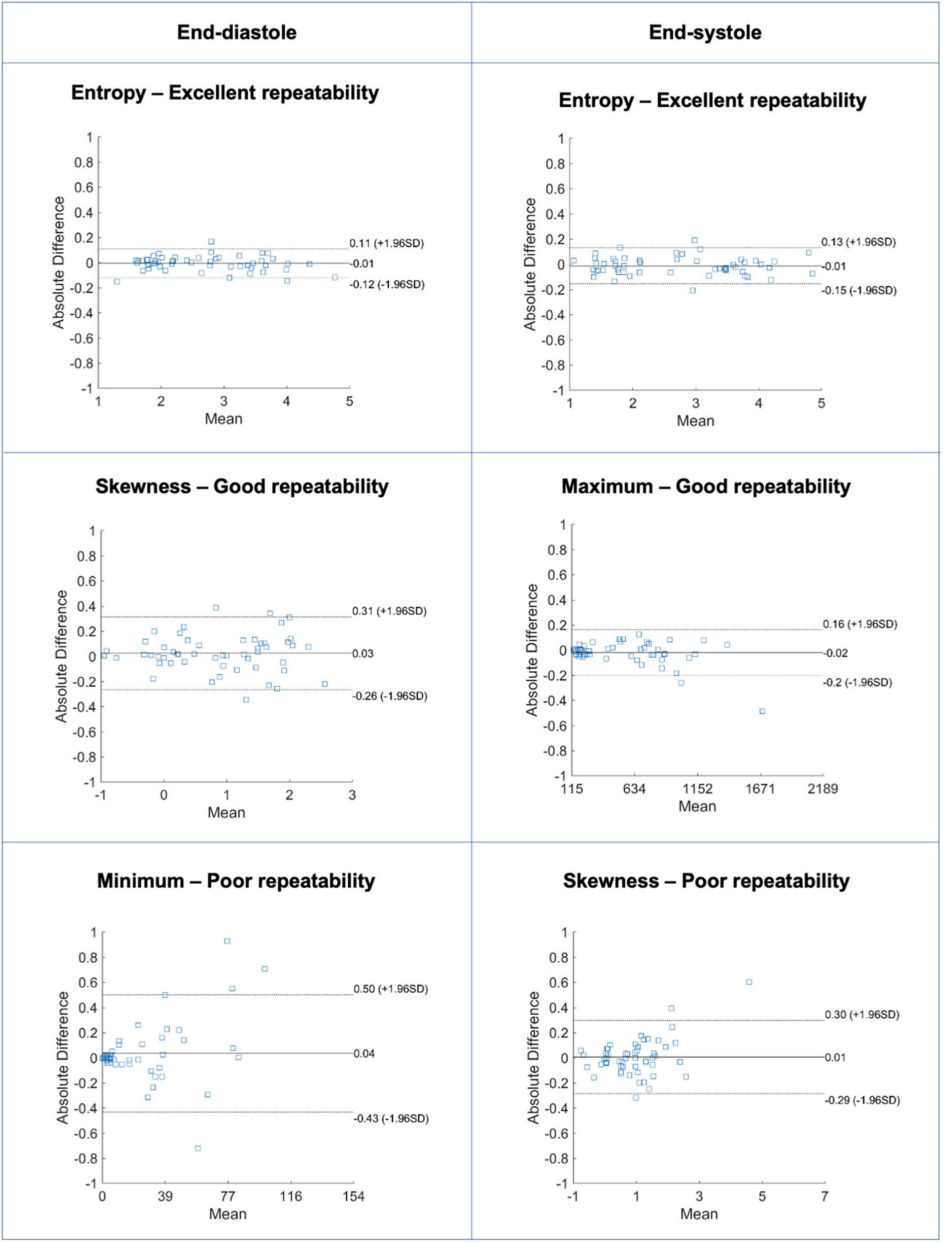
Bland-Altman plots for selected LV myocardium first-order features in end-diastole (left) and end-systole (right) with different levels of repeatability. Differences in Bland-Altman are calculated after normalizing radiomics in the range [0–1] to facilitate comparison among different features. All features are unitless. LV: left ventricle.

**Figure 7:**
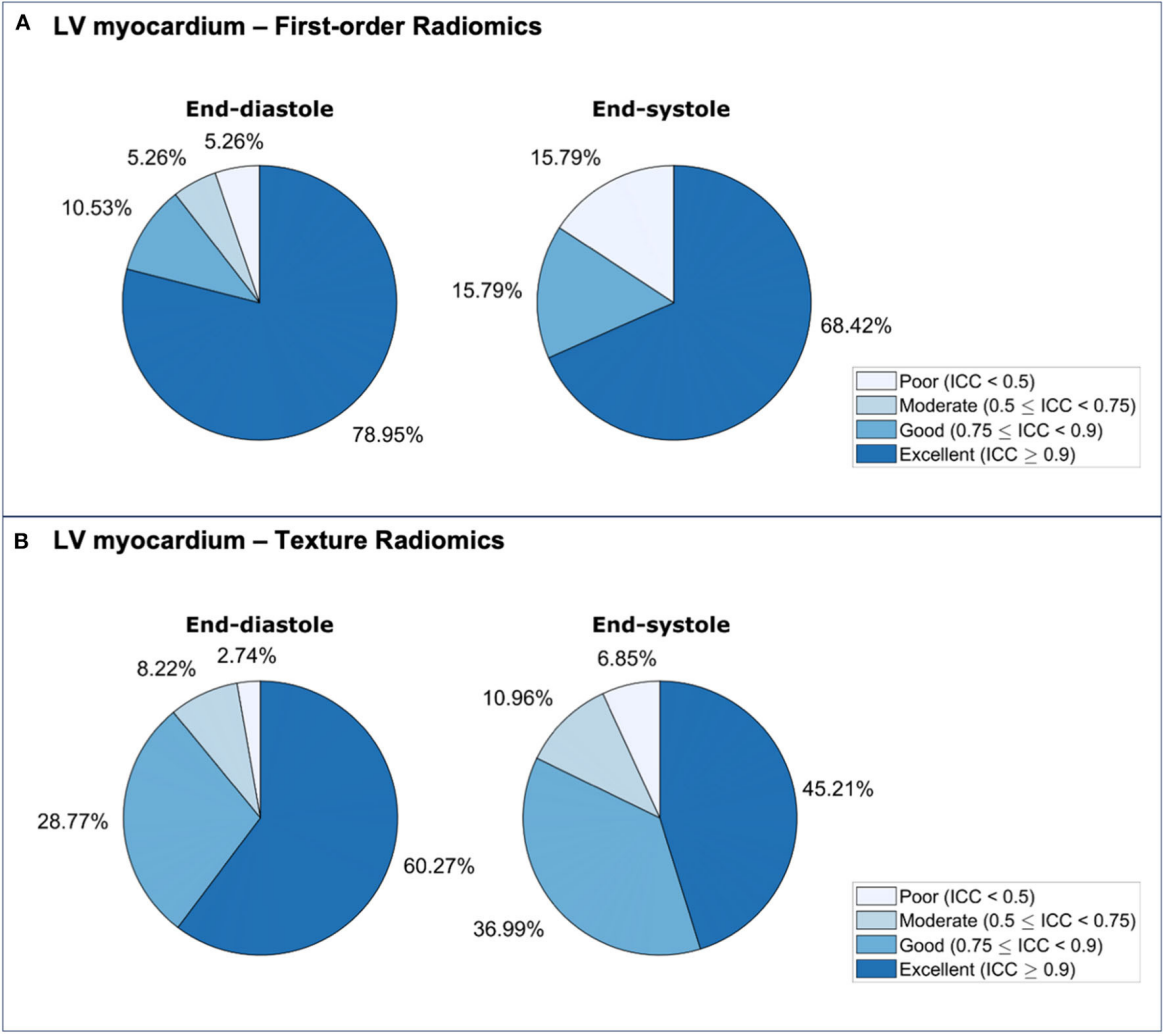
Repeatability of LV myocardium radiomics first-order **(A)** and texture **(B)** features in end-diastole and end-systole. ICC: intra-class correlation coefficient; LV: left ventricle.

Repeatability of LV myocardium first-order features varied from poor to excellent with mean ICC ranging from 0.333 to 0.964 [Median (IQR): 0.932 (0.140)] (Table 5, Supplementary Table 5, Figure 6). The proportion of features demonstrating excellent repeatability (28/38, 74%) was substantially higher than that seen for the shape features. This was alongside a small number (4/38, 11%) of particularly poorly performing features. Overall, repeatability was high in both end-diastole and end-systole, with marginally better overall performance in the former (Figure 7A). For both cardiac phases, the best performing first-order features were “entropy,” “percentile 90,” “root mean squared,” “median,” and “mean.” The following features had the worst performance in both end-diastole and end-systole: “kurtosis,” “minimum,” “skewness,” and “variance.”

**Table 5:** Repeatability of left ventricular myocardium first-order features in end-diastole.

**Feature name**	**Robustness**	**ICC (95% CI)**	**CV (%)**	**MRD (%)**
Entropy	Excellent	0.962 (0.936, 0.978)	8.9	9.7
90th percentile	Excellent	0.961 (0.934, 0.977)	11.9	11.8
Root mean squared	Excellent	0.959 (0.930, 0.976)	11.9	11.4
Median	Excellent	0.958 (0.928, 0.975)	12.4	11.9
Mean	Excellent	0.957 (0.927, 0.975)	12.1	11.5
Energy	Excellent	0.950 (0.915, 0.970)	25.2	27.1
Uniformity	Excellent	0.942 (0.902, 0.966)	13.0	14.0
Mean absolute deviation	Excellent	0.934 (0.890, 0.961)	15.1	16.3
10th percentile	Excellent	0.933 (0.888, 0.961)	15.0	15.0
Robust mean absolute deviation	Excellent	0.932 (0.885, 0.960)	15.5	16.5
Interquartile range	Excellent	0.929 (0.881, 0.958)	15.4	15.9
Standard deviation	Excellent	0.918 (0.864, 0.952)	15.8	17.3
Total energy	Excellent	0.912 (0.853, 0.948)	26.0	28.0
Maximum	Good	0.875 (0.794, 0.925)	19.1	21.0
Range	Good	0.810 (0.694, 0.885)	20.8	23.4
Variance	Good	0.802 (0.683, 0.880)	30.4	33.7
Skewness	Poor	0.434 (0.192, 0.627)	187.5	72.7
Minimum	Poor	0.401 (0.154, 0.602)	62.1	65.9
Kurtosis	Poor	0.369 (0.116, 0.577)	39.3	41.5

*CI, confidence interval; CV, coefficient of variation; ICC, intra-class correlation coefficient; MRD, mean relative difference*.

### Repeatability of LV Myocardium Texture Features

**Figure 8:**
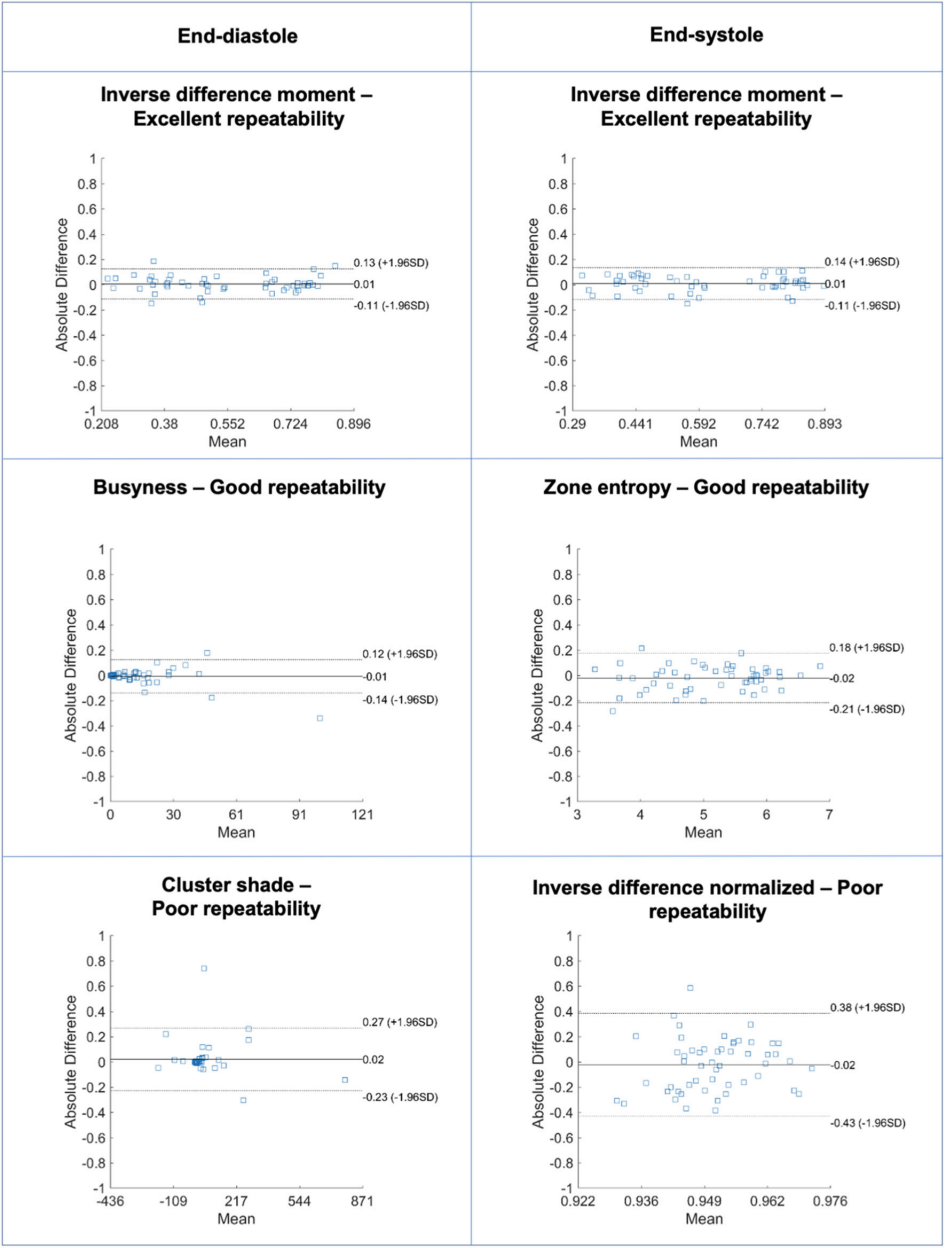
Bland-Altman plots for selected LV myocardium texture features in end-diastole (left) and end-systole (right) with different levels of repeatability. Differences in Bland-Altman are calculated after normalizing radiomics in the range [0–1] to facilitate comparison among different features. All features are unitless. LV: left ventricle.

Repeatability of LV myocardium texture features varied from poor to excellent with mean ICC ranging from −0.130 to 0.977 [Median (IQR): 0.907 (0.006)] (Supplementary Tables 6, 7, Figure 8). The majority of texture features had good or excellent repeatability (125/146, 86%). A small minority of features had poor repeatability (7/146, 4.8%). There was slightly better repeatability in end-diastole than in end-systole (Figure 7B). We present the ten best and worst performing texture feature and their corresponding ICCs in end-diastole (Table 6) and end-systole (Supplementary Table 8). Across both end-diastole and end-systole, “cluster shade” and “cluster prominence” were poorly performing features. In end-systole, “strength,” “inverse difference normalized,” and “inverse difference moment normalized” also demonstrated poor repeatability.

**Table 6:** The 10 most and 10 least robust left ventricular myocardium texture features in end-diastole.

**Feature name**	**Robustness**	**ICC (95% CI)**	**CV (%)**	**MRD (%)**
Inverse difference moment	Excellent	0.975 (0.957, 0.985)	6.94	6.48
Inverse difference	Excellent	0.973 (0.955, 0.984)	5.05	4.82
Joint entropy	Excellent	0.973 (0.953, 0.984)	7.79	7.24
Run length non uniformity normalized	Excellent	0.970 (0.949, 0.983)	4.45	4.10
Short run emphasis	Excellent	0.970 (0.948, 0.982)	2.18	1.99
Difference entropy	Excellent	0.965 (0.940, 0.979)	7.48	7.54
Run percentage	Excellent	0.963 (0.938, 0.979)	3.84	3.17
Small dependence emphasis	Excellent	0.960 (0.933, 0.977)	11.69	11.87
Sum entropy	Excellent	0.959 (0.931, 0.976)	7.22	6.77
Sum average	Excellent	0.958 (0.930, 0.976)	11.03	11.7
Gray level variance	Good	0.792 (0.668, 0.874)	28.66	31.84
Informal measure of correlation 2	Good	0.755 (0.612, 0.850)	11.91	12.33
Complexity	Moderate	0.744 (0.597, 0.843)	38.65	42.09
Inverse difference normalized	Moderate	0.720 (0.563, 0.827)	0.72	0.80
Strength	Moderate	0.717 (0.559, 0.825)	40.74	47.21
Informal measure of correlation 1	Moderate	0.695 (0.528, 0.811)	20.64	21.63
Inverse difference moment normalized	Moderate	0.676 (0.502, 0.798)	0.23	0.24
Correlation	Moderate	0.562 (0.350, 0.720)	19.12	20.66
Cluster shade	Poor	0.420 (0.175, 0.616)	204.88	74.52
Cluster prominence	Poor	0.364 (0.110, 0.573)	60.66	69.95

*CI, confidence interval; CV, coefficient of variation; ICC, intra-class correlation coefficient; MRD, mean relative difference*.

We also evaluated differences in the reproducibility of features by texture class i.e., GLCM, GLRLM, GLSZM, NGTDM, and GLDM (Supplementary Figure 2). The most striking difference between texture classes was the variation in the range of ICC values. The GLCM class had the widest ICC range with very low ICC values calculated for some of the features in this class. Indeed, six of the seven texture features with the poorest repeatability belong to the GLCM class. However, broadly, all texture classes had similar mean repeatability; with the exception of GLRLM that had a significantly greater average repeatability than NGTDM, no other pairs of classes showed a significant difference in mean ICC.

## Discussion

### Summary of Findings

In this heterogenous case mix of test-retest studies, we demonstrated wide variation in the repeatability of CMR radiomics features by ROI, feature category and cardiac phase. There were features with good and excellent repeatability within all feature categories and ROIs. The signal intensity-based features (first-order, texture) demonstrated the greatest variation in repeatability comprising a large proportion of highly reproducible features alongside features with the poorest repeatability. We present details of repeatability performance for a comprehensive range of radiomics features, which is intended to guide selection of the most robust features for clinical modeling by future researchers. Therefore, this work is an important step in characterizing the technical performance of CMR radiomics and enhancing future efforts to evaluate its clinical utility.

### Comparison With Existing Literature

There have been recent efforts to define the repeatability of radiomics features relating to oncological imaging with test-retest studies (21–23) and using phantom (24), image translation (25), and image pertubation (26) experiments. These studies demonstrate variation in feature repeatability and emphasize the need to actively seek and select robust features for modeling purposes. However, these findings have limited transferability to CMR radiomics, due to the modalities studied (mostly CT) and because the ROIs selected for oncological tumor analysis are not comparable to those typically selected for CMR analysis. Nevertheless, our findings of variation in repeatability by feature category (first-order > shape > textural) are in close agreement with previous work regarding cancer radiomics.

Jang et al. (27) present the only other study to consider repeatability of CMR radiomics LV texture features (rather than texture, first order, and shape features in our analysis) in 51 patients with clinical indication for CMR scanned twice in the same session with a 3 Tesla Siemens scanner. A subset of the study participants had abnormal CMR findings (“normal” *n* = 14, non-ischaemic cardiomyopathy *n* = 16, ischaemic cardiomyopathy *n* = 5, hypertrophic cardiomyopathy *n* = 2, other *n* = 14). The authors report variation in repeatability between classes of texture features and, similar to our findings, demonstrate that only a subset has high repeatability. Overall, when comparing equivalent measures of intra-observer variability for LV texture features, we had better repeatability indices compared to that reported by Jang et al. (27). This may reflect differences in contouring SOP between the two approaches; our contouring methodology is designed to avoid blood pool or pericardial fat in myocardial contours as inclusion of these in analysis can highly distort texture feature values, it is not clear if this was a key part of the SOP used by Jang et al. (27). Whilst we include both 1.5 and 3 Tesla scanners in the sample, the majority of our cases were scanned with a 1.5 Tesla scanner. 3 Tesla sequences are more prone to artifacts specially dark/bright lines across images and this too may have contributed to the poorer repeatability observed by Jang et al. (27). Studies in larger samples are warranted to further explore potential explanations for these differences and to perform subgroup analyses.

Our study is the first to report repeatability of LV and RV CMR radiomics shape features. Radiomics shape features are calculated from 3D image masks derived from image contours, as such, their repeatability is a direct reflection of segmentation robustness. For instance, we demonstrate better repeatability of features quantifying the heart short axis, e.g., “least axis length,” “minor axis length” and “maximal 2D diameter,” than those quantifying the long axis, e.g., “major axis length” and “maximum 3D diameter.” The reduced reproducibility of features along the cardiac long axis likely reflects segmentation robustness which is likely to suffer more at the apex and base of the heart rather than in the middle slices. This is consistent with our observation of low repeatability of all features quantifying ventricular sphericity.

Signal intensity-based features (first-order, texture) applied to the LV myocardium reflect both segmentation and signal intensities within the defined ROI. These features are therefore sensitive to variations in image acquisition which affect intensity levels within the whole image. Furthermore, there is potential to introduce extreme outlier values in the segmentation process. For instance, an LV endocardial contour that is not perfectly opposed to the endocardium would introduce a series of high value voxels from the blood pool into what will be defined as “myocardium” for radiomics analysis (Supplementary Figure 3). Our findings support these theoretical suppositions. The most reproducible first-order features within the LV myocardium (“entropy,” “root mean squared,” “median,” “mean,”) are measures of the average voxel SI levels, whilst the least reproducible first-order features (“kurtosis,” “minimum,” “skewness,” “variance”) are measures of their spread. Consistent with this, the least reproducible texture features, “cluster shade” and “cluster prominence,” also represent measures of skewness.^30^ These measures of spread are, of course, more susceptible to small variations in extreme signal intensity values. Notably, repeatability of conventional CMR indices in our study exceeded that of published reports. Particularly, the metric most relevant for defining the LV myocardium for LV analysis, LV mass, had excellent repeatability with ICC of 0.95 (0.91, 0.97). Therefore, as would be expected, radiomics features have, in general, much higher sensitivity to small variations in segmentation, which appear inconsequential to conventional metrics. Texture radiomics are affected not only by segmentation but are additionally sensitive to image acquisition settings and pre-processing. Variation in image signal intensities due to technical factors (scanner specifications, sequence acquisition parameters) may be reduced through pre-processing intensity normalization techniques, which may improve the repeatability of signal intensity-based radiomics by “smoothing” variations in intensity levels.

### Study Limitations and Directions for Future Research

This study presents an important first step in evaluating the technical performance of CMR radiomics first-order, texture, and shape feature. The present dataset does not permit consideration of the wide range of technical and population related factors that may be modifying the repeatability performance of radiomics features. Studies considering the impact of factors such as scanner vendor/model, magnet strength, acquisition parameters, and disease are warranted. To guide building of radiomics models that would truly translate to clinical practice, we should consider robustness of features not only under repeatability, but also under reproducibility conditions, where real-life variations in scanner, operator, and image acquisition are not strictly controlled. Finally, different technical approaches to feature extraction and image normalization may improve robustness of radiomics features, in particular for intensity-based features. For example, different approaches to gray level discretisation have been shown to affect feature robustness (28) and future research on optimizing bin width or bin number may improve radiomics robustness. Lastly, we have focused on radiomics computed on original (untransformed) images. Whilst this covers the vast majority of features in common use, there are additional features that are beyond the scope of this study, such as features extracted from mathematical transformations of the original images. There is also need for study of normalization techniques which may improve repeatability performance of radiomics features; this is a broad topic with a large number of normalization options (e.g., histogram matching, generative adversarial networks) that should be considered systematically in dedicated studies.

## Conclusions

There is variation in the repeatability of CMR radiomics features, which is likely to be clinically relevant. In this paper we present repeatability performance of a comprehensive range of commonly used CMR radiomics features. The work is intended to guide future researchers to select the most robust radiomics features for clinical modeling. Further work in larger and richer datasets and experimentation with different technical approaches is needed to further define the repeatability and reproducibility of CMR radiomics and to ascertain the optimal technical approach for radiomics analysis for maintaining feature robustness.

## Data Availability Statement

Publicly available datasets were analyzed in this study. This data can be found here: https://thevolumesresource.com.

## Author Contributions

ZR-E, SEP, KL, NCH, and PBM conceived the study. ZR-E and PG wrote the manuscript. ZR-E and SEP analyzed the CMR scans. JC supervised and advised on the statistical analysis. PG extracted radiomics features and conducted the statistical analysis. AJ contributed to manuscript editing and statistical analysis. JA, ANB, RHD, CHM, and JCM collated the studies in the VOLUMES resource. All authors provided critical review of the manuscript.

## Conflict of Interest

The authors declare that the research was conducted in the absence of any commercial or financial relationships that could be construed as a potential conflict of interest.
