# Supplementary material for: Repeatability of Cardiac Magnetic Resonance Radiomics: A Multi-Centre Multi-Vendor Test-Retest Study
Source: Front Cardiovasc Med. 2020 Dec 2;7:586236. doi: 10.3389/fcvm.2020.586236 (PMC7738466; doi:10.3389/fcvm.2020.586236)
Supplement: Supplementary file 1 [file Table_1.DOCX]

**Supplementary Table 1 Typical sequence and scanner parameters*.**

| Typical sequence parameters | Centre 1 | Centre 2 | Centre 3 | Centre 4 | Centre 5 |
| --- | --- | --- | --- | --- | --- |
| n | 24 | 35 | 31 | 15 | 5 |
| Manufacturer | Siemens Healthcare | Siemens Healthcare | Siemens Healthcare | Philips Medical | Siemens Healthcare |
| Model | Avanto | Aera | Avanto | Achieva | Avanto |
| Strength/ Tesla | 1.5 | 1.5 | 1.5 | 3 | 1.5 |
| Slice thickness/ mm | 7 | 8 | 8 | 10 | 7 |
| Gap/ mm | 3 | 2 | 0 | 0 | 3 |
| Cardiac phases | 25 | 25 | 25 | 30 | 25 |
| Field of view/ cm | 270 $\times$360 | 340$\times420$ | 308$\times$380 | 288$\times$288 | 270$\times$340 |
| Acquisition matrix | 320$\times$168 | 208$\times$153 | 256$\times$208 | 153$\times$158 | 208$\times$170 |
| Echo time/ ms | 1.43 | 1.07 | 1.21 | 1.28 | 1.17 |
| Temporal resolution/ ms | 50.3 | 33.2 | 51.7 | 36.6 | 46.6 |
| Flip angle/ degrees | 80 | 74 | 78 | 40 | 67 |
| Bandwidth/ Hz/pixel | 919 | 925 | 930 | 2569 | 962 |

**Supplementary Table 1 footnote:** Hz = hertz; mm = millimeters; ms = milliseconds. * Adapted from: Bhuva AN, Bai W, Lau C, Davies R, Ye Y, Bulluck H, McAlindon E, Culotta V, Swoboda P, Captur G, Treibel T, Augusto J, Knott K, Seraphim A, Cole G, Petersen S, Edwards N, Greenwood J, Bucciarelli-Ducci C, Hughes A, Rueckert D, Moon JC, Manisty C. A Multicenter, Scan-Rescan, Human and Machine Learning CMR Study to Test Generalizability and Precision in Imaging Biomarker Analysis. Circ Cardiovasc Imaging 2019 Oct 1;12(10):e009214.

**Supplementary Table 2. Repeatability for conventional CMR indices (in descending order of robustness)**

| CMR indices | Robustness | ICC (95% CI) | CV (%) | MRD (%) |
| --- | --- | --- | --- | --- |
| LV end-systolic volume | Excellent | 0.97 (0.96, 0.99) | 8.41 | 9.47 |
| LV end-diastolic volume | Excellent | 0.96 (0.93, 0.98) | 5.35 | 5.58 |
| LV mass | Excellent | 0.95 (0.91, 0.97) | 7.34 | 8.6 |
| RV end-systolic volume | Excellent | 0.94 (0.90, 0.97) | 12.38 | 14.51 |
| RV end-diastolic volume | Good | 0.89 (0.83, 0.94) | 11.03 | 11.52 |
| LV ejection fraction | Good | 0.89 (0.82, 0.94) | 7.05 | 7.13 |
| RV ejection fraction | Moderate | 0.66 (0.48, 0.79) | 13.18 | 14.55 |

**Supplementary Table 2 footnote:** CI: confidence interval; CV: Coefficient of variation; ICC: intra-class correlation coefficient; LV: left ventricle; MRD: Mean relative difference; RV: right ventricle

**Supplementary Table 3**. **Repeatability of left ventricular blood pool shape features in end-systole**

| Feature name | Robustness | ICC (95% CI) | CV (%) | MRD (%) |
| --- | --- | --- | --- | --- |
| Volume | Excellent | 0.974 (0.957, 0.985) | 8.41 | 9.47 |
| Least axis length | Excellent | 0.940 (0.900, 0.965) | 4.00 | 4.31 |
| Surface area | Excellent | 0.932 (0.885, 0.960) | 7.61 | 7.99 |
| Surface area to volume ratio | Excellent | 0.919 (0.865, 0.952) | 4.16 | 4.36 |
| Minor axis length | Good | 0.860 (0.770, 0.916) | 5.99 | 5.29 |
| Major axis length | Good | 0.848 (0.752, 0.909) | 6.08 | 6.62 |
| Maximum 2D diameter (Slice) | Good | 0.831 (0.727, 0.898) | 7.07 | 7.18 |
| Flatness | Good | 0.830 (0.724, 0.897) | 7.32 | 7.73 |
| Elongation | Good | 0.791 (0.666, 0.873) | 8.31 | 9.04 |
| Maximum 2D diameter (Column) | Good | 0.779 (0.649, 0.865) | 5.61 | 5.58 |
| Maximum 2D diameter (Row) | Good | 0.752 (0.608, 0.848) | 6.19 | 6.06 |
| Maximum 3D diameter | Moderate | 0.725 (0.570, 0.830) | 6.40 | 6.54 |
| Compactness 2 | Moderate | 0.691 (0.522, 0.808) | 11.46 | 11.16 |
| Compactness | Moderate | 0.673 (0.497, 0.796) | 5.81 | 5.61 |
| Sphericity | Moderate | 0.666 (0.487, 0.791) | 3.88 | 3.75 |
| Spherical disproportion | Moderate | 0.630 (0.440, 0.767) | 3.88 | 3.75 |

**Supplementary Table 3 footnote:** CI: confidence interval; CV: Coefficient of variation; ICC: intra-class correlation coefficient; MRD: Mean relative difference

**Supplementary Table 4. Repeatability of right ventricular blood pool shape features in end-systole**

| Feature name | Robustness | ICC (95% CI) | CV (%) | MRD (%) |
| --- | --- | --- | --- | --- |
| Volume | Excellent | 0.941 (0.900, 0.965) | 12.38 | 14.51 |
| Surface area | Excellent | 0.934 (0.889, 0.961) | 8.52 | 9.94 |
| Surface area to volume ratio | Good | 0.888 (0.814, 0.933) | 6.7 | 7.56 |
| Minor axis length | Good | 0.835 (0.733, 0.901) | 9.61 | 11.03 |
| Maximum 2D diameter (Slice) | Good | 0.834 (0.731, 0.900) | 5.54 | 5.83 |
| Maximum 3D diameter | Good | 0.830 (0.724, 0.897) | 5.19 | 5.63 |
| Least axis length | Good | 0.820 (0.709, 0.891) | 6.56 | 6.72 |
| Maximum 2D diameter (Row) | Good | 0.819 (0.707, 0.890) | 6.42 | 6.60 |
| Maximum 2D diameter (Column) | Good | 0.782 (0.652, 0.867) | 9.37 | 9.93 |
| Major axis length | Moderate | 0.740 (0.592, 0.840) | 6.08 | 6.8 |
| Elongation | Moderate | 0.634 (0.444, 0.770) | 12.74 | 14.66 |
| Flatness | Moderate | 0.621 (0.426, 0.760) | 8.72 | 9.33 |
| Spherical disproportion | Moderate | 0.595 (0.393, 0.743) | 4.38 | 4.97 |
| Sphericity | Moderate | 0.575 (0.367, 0.729) | 4.38 | 4.97 |
| Compactness | Moderate | 0.570 (0.360, 0.725) | 6.56 | 7.44 |
| Compactness 2 | Moderate | 0.556 (0.342, 0.715) | 13.02 | 14.82 |

**Supplementary Table 4 footnote:** CI: confidence interval; CV: Coefficient of variation; ICC: intra-class correlation coefficient; MRD: Mean relative difference

**Supplementary Table 5. Repeatability of left ventricular myocardium shape features in end-systole**

| Feature name | Robustness | ICC (95% CI) | CV (%) | MRD (%) |
| --- | --- | --- | --- | --- |
| Minor axis length | Excellent | 0.960 (0.933, 0.977) | 2.44 | 2.30 |
| Least axis length | Excellent | 0.931 (0.885, 0.959) | 3.55 | 3.56 |
| Surface area | Excellent | 0.929 (0.881, 0.958) | 6.04 | 6.43 |
| Volume | Excellent | 0.912 (0.853, 0.948) | 9.84 | 10.42 |
| Maximum 2D diameter (Slice) | Good | 0.899 (0.833, 0.940) | 3.52 | 3.41 |
| Surface Area to Volume Ratio | Good | 0.856 (0.765, 0.914) | 8.11 | 8.6 |
| Spherical disproportion | Good | 0.853 (0.761, 0.912) | 5.84 | 6.24 |
| Major axis length | Good | 0.850 (0.756, 0.910) | 4.66 | 4.87 |
| Compactness 2 | Good | 0.844 (0.746, 0.906) | 17.21 | 18.51 |
| Sphericity | Good | 0.841 (0.742, 0.904) | 5.84 | 6.24 |
| Compactness | Good | 0.841 (0.742, 0.904) | 8.73 | 9.34 |
| Maximum 2D diameter (Row) | Good | 0.837 (0.736, 0.902) | 4.90 | 5.22 |
| Elongation | Good | 0.776 (0.644, 0.863) | 5.23 | 5.26 |
| Maximum 2D diameter (Column) | Moderate | 0.740 (0.591, 0.840) | 5.57 | 5.00 |
| Maximum 3D diameter | Moderate | 0.728 (0.575, 0.832) | 5.96 | 5.37 |
| Flatness | Moderate | 0.707 (0.545, 0.819) | 5.66 | 5.32 |

**Supplementary Table 5 footnote:** CI: confidence interval; CV: Coefficient of variation; ICC: intra-class correlation coefficient; MRD: Mean relative difference

**Supplementary Table 6**. **Repeatability of left ventricular myocardium first-order features in end-systole**

| Feature name | Robustness | ICC (95% CI) | CV (%) | MRD (%) |
| --- | --- | --- | --- | --- |
| Entropy | Excellent | 0.964 (0.939, 0.979) | 7.87 | 7.36 |
| 90^th^ Percentile | Excellent | 0.963 (0.938, 0.978) | 11.43 | 11.27 |
| Mean | Excellent | 0.960 (0.932, 0.977) | 11.94 | 11.64 |
| Root mean squared | Excellent | 0.959 (0.931, 0.976) | 11.82 | 11.69 |
| Median | Excellent | 0.956 (0.926, 0.974) | 12.58 | 12.25 |
| 10^th^ Percentile | Excellent | 0.953 (0.920, 0.972) | 14.86 | 15.33 |
| Energy | Excellent | 0.943 (0.905, 0.967) | 24.01 | 24.97 |
| Uniformity | Excellent | 0.941 (0.901, 0.965) | 13.18 | 13.86 |
| Interquartile range | Excellent | 0.939 (0.898, 0.964) | 14.67 | 14.95 |
| Robust mean absolute deviation | Excellent | 0.931 (0.884, 0.959) | 14.41 | 15.02 |
| Mean absolute deviation | Excellent | 0.926 (0.877, 0.956) | 14.56 | 15.78 |
| Maximum | Excellent | 0.922 (0.870, 0.954) | 16.15 | 16.87 |
| Range | Excellent | 0.918 (0.864, 0.952) | 16.9 | 17.69 |
| Total energy | Excellent | 0.918 (0.863, 0.951) | 24.84 | 25.71 |
| Standard deviation | Excellent | 0.913 (0.855, 0.949) | 15.58 | 17.06 |
| Skewness | Good | 0.802 (0.683, 0.880) | 87.38 | 44.97 |
| Variance | Good | 0.792 (0.668, 0.874) | 29.96 | 33.25 |
| Kurtosis | Moderate | 0.535 (0.315, 0.700) | 25.89 | 28.63 |
| Minimum | Poor | 0.333 (0.075, 0.549) | NA | NA |

**Supplementary Table 6 footnote:** CI: confidence interval; CV: Coefficient of variation; ICC: intra-class correlation coefficient; MRD: Mean relative difference; NA: not available. NA corresponds to cases where robustness indices were not available metric due to radiomics feature value of zero.

**Supplementary Table 7**. **Repeatability of LV myocardium texture features in end-diastole**

| Feature name | Texture class | Robustness | ICC (95% CI) | CV (%) | MRD (%) |
| --- | --- | --- | --- | --- | --- |
| Inverse difference moment | GLCM | Excellent | 0.975 (0.957, 0.985) | 6.94 | 6.48 |
| Inverse difference | GLCM | Excellent | 0.973 (0.955, 0.984) | 5.05 | 4.82 |
| Joint entropy | GLCM | Excellent | 0.973 (0.953, 0.984) | 7.79 | 7.24 |
| Run length non uniformity normalized | GLRLM | Excellent | 0.970 (0.949, 0.983) | 4.45 | 4.10 |
| Short run emphasis | GLRLM | Excellent | 0.970 (0.948, 0.982) | 2.18 | 1.99 |
| Difference entropy | GLCM | Excellent | 0.965 (0.940, 0.979) | 7.48 | 7.54 |
| Run percentage | GLRLM | Excellent | 0.963 (0.938, 0.979) | 3.84 | 3.17 |
| Small dependence emphasis | GLDM | Excellent | 0.960 (0.933, 0.977) | 11.69 | 11.87 |
| Sum entropy | GLCM | Excellent | 0.959 (0.931, 0.976) | 7.22 | 6.77 |
| Sum average | GLCM | Excellent | 0.958 (0.930, 0.976) | 11.03 | 11.7 |
| Joint average | GLCM | Excellent | 0.958 (0.930, 0.976) | 11.03 | 11.7 |
| Zone percentage | GLSZM | Excellent | 0.957 (0.928, 0.975) | 13.65 | 13.98 |
| Low grey level zone emphasis | GLSZM | Excellent | 0.953 (0.921, 0.973) | 19.75 | 22.3 |
| Low grey level run emphasis | GLRLM | Excellent | 0.953 (0.921, 0.972) | 20.8 | 23.31 |
| Short run low grey level emphasis | GLRLM | Excellent | 0.951 (0.918, 0.971) | 20.66 | 22.97 |
| Low grey level emphasis | GLDM | Excellent | 0.950 (0.916, 0.971) | 21.18 | 23.66 |
| Grey level non uniformity | GLRLM | Excellent | 0.947 (0.910, 0.969) | 14.97 | 15.31 |
| Grey level non uniformity normalized | GLRLM | Excellent | 0.946 (0.910, 0.968) | 12.26 | 12.58 |
| Small area low grey level emphasis | GLSZM | Excellent | 0.941 (0.900, 0.965) | 20.29 | 22.93 |
| Small area emphasis | GLSZM | Excellent | 0.938 (0.896, 0.963) | 3.49 | 3.75 |
| Difference average | GLCM | Excellent | 0.934 (0.889, 0.961) | 14.27 | 14.31 |
| Dependence variance | GLDM | Excellent | 0.933 (0.888, 0.961) | 19.5 | 20.05 |
| Size zone non uniformity normalized | GLSZM | Excellent | 0.932 (0.886, 0.960) | 6.32 | 6.83 |
| Large dependence emphasis | GLDM | Excellent | 0.931 (0.885, 0.960) | 17.65 | 17.83 |
| Grey level non uniformity | GLDM | Excellent | 0.929 (0.881, 0.958) | 16.47 | 17.73 |
| Dependence non uniformity normalized | GLDM | Excellent | 0.929 (0.881, 0.958) | 10.2 | 10.01 |
| Long run high grey level emphasis | GLRLM | Excellent | 0.925 (0.875, 0.956) | 20.45 | 21.1 |
| High grey level zone emphasis | GLSZM | Excellent | 0.924 (0.872, 0.955) | 19.16 | 21.53 |
| Run length non uniformity | GLRLM | Excellent | 0.924 (0.872, 0.955) | 12.89 | 13.12 |
| Autocorrelation | GLCM | Excellent | 0.922 (0.870, 0.954) | 21.6 | 23.1 |
| Grey level non uniformity | GLSZM | Excellent | 0.920 (0.866, 0.952) | 15 | 15.15 |
| Size zone non uniformity | GLSZM | Excellent | 0.920 (0.866, 0.952) | 15 | 15.15 |
| High grey level run emphasis | GLRLM | Excellent | 0.920 (0.866, 0.952) | 20.66 | 22.26 |
| High grey level emphasis | GLDM | Excellent | 0.919 (0.865, 0.952) | 20.72 | 22.1 |
| Large dependence low grey level emphasis | GLDM | Excellent | 0.918 (0.863, 0.951) | 30.6 | 32.14 |
| Coarseness | NGTDM | Excellent | 0.917 (0.861, 0.951) | 13.68 | 14.11 |
| Short run high grey level emphasis | GLRLM | Excellent | 0.916 (0.860, 0.950) | 21.26 | 22.65 |
| Run entropy | GLRLM | Excellent | 0.916 (0.860, 0.950) | 4.06 | 4.28 |
| Dependence non uniformity | GLDM | Excellent | 0.911 (0.852, 0.947) | 13.01 | 13.68 |
| Maximum probability | GLCM | Excellent | 0.911 (0.852, 0.947) | 23.17 | 25.15 |
| Zone entropy | GLSZM | Excellent | 0.909 (0.848, 0.946) | 3.49 | 3.33 |
| Long run low grey level emphasis | GLRLM | Excellent | 0.906 (0.844, 0.945) | 23.97 | 25.71 |
| Small area high grey level emphasis | GLSZM | Excellent | 0.905 (0.842, 0.943) | 21.03 | 23.47 |
| Contrast | NGTDM | Excellent | 0.901 (0.836, 0.941) | 22.28 | 25.62 |
| Busyness | NGTDM | Good | 0.899 (0.833, 0.940) | 26.81 | 31.44 |
| Small dependence low grey level emphasis | GLDM | Good | 0.899 (0.833, 0.940) | 20.74 | 22.29 |
| Joint energy | GLCM | Good | 0.884 (0.809, 0.931) | 22.25 | 24.17 |
| Long run emphasis | GLRLM | Good | 0.883 (0.808, 0.930) | 9.46 | 8.19 |
| Run variance | GLRLM | Good | 0.872 (0.790, 0.924) | 17.42 | 17.31 |
| Large dependence high grey level emphasis | GLDM | Good | 0.871 (0.788, 0.923) | 22.03 | 24.68 |
| Large area high grey level emphasis | GLSZM | Good | 0.858 (0.768, 0.915) | 40.63 | 41.51 |
| Contrast | GLCM | Good | 0.852 (0.759, 0.911) | 26.14 | 27.91 |
| Large area emphasis | GLSZM | Good | 0.847 (0.751, 0.908) | 45.48 | 47.38 |
| Zone variance | GLSZM | Good | 0.847 (0.751, 0.908) | 47.33 | 49.37 |
| Dependence entropy | GLDM | Good | 0.840 (0.741, 0.904) | 2.56 | 2.71 |
| Small dependence high grey level emphasis | GLDM | Good | 0.836 (0.734, 0.901) | 27.14 | 29.36 |
| Difference variance | GLCM | Good | 0.835 (0.732, 0.901) | 26.78 | 28.62 |
| Gray level variance | GLSZM | Good | 0.832 (0.729, 0.899) | 25.23 | 27.65 |
| Inverse variance | GLCM | Good | 0.821 (0.710, 0.892) | 8.44 | 7.13 |
| Sum of squares | GLCM | Good | 0.819 (0.708, 0.891) | 27.34 | 30.21 |
| Cluster tendency | GLCM | Good | 0.800 (0.680, 0.879) | 29.40 | 32.98 |
| Gray level variance | GLRLM | Good | 0.795 (0.671, 0.875) | 28.07 | 30.81 |
| Large area low grey level emphasis | GLSZM | Good | 0.793 (0.669, 0.874) | 52.46 | 54.53 |
| Gray level variance | GLDM | Good | 0.792 (0.668, 0.874) | 28.66 | 31.84 |
| Informal measure of correlation2 | GLCM | Good | 0.755 (0.612, 0.850) | 11.91 | 12.33 |
| Complexity | NGTDM | Moderate | 0.744 (0.597, 0.843) | 38.65 | 42.09 |
| Inverse difference normalized | GLCM | Moderate | 0.720 (0.563, 0.827) | 0.72 | 0.8 |
| Strength | NGTDM | Moderate | 0.717 (0.559, 0.825) | 40.74 | 47.21 |
| Informal measure of correlation1 | GLCM | Moderate | 0.695 (0.528, 0.811) | 20.64 | 21.63 |
| Inverse difference moment normalized | GLCM | Moderate | 0.676 (0.502, 0.798) | 0.23 | 0.24 |
| Correlation | GLCM | Moderate | 0.562 (0.350, 0.720) | 19.12 | 20.66 |
| Cluster shade | GLCM | Poor | 0.420 (0.175, 0.616) | 204.88 | 74.52 |
| Cluster prominence | GLCM | Poor | 0.364 (0.110, 0.573) | 60.66 | 69.95 |

**Supplementary Table 7 footnote:** CI: confidence interval; CV: coefficient of variation; GLCM: grey level co-occurrence matrix; GLDM: grey level dependence matrix; GLRLM: grey level run length matrix; GLSZM: grey level size zone matrix; NGTDM: neighbouring grey tone difference matrix; ICC:

intra-class correlation coefficient; LV: left ventricle; MRD: mean relative difference

**Supplementary Table 8**. **Repeatability of LV myocardium texture features in end-systole**

| Feature name | Class | Robustness | ICC (95% CI) | CV (%) | MRD (%) |
| --- | --- | --- | --- | --- | --- |
| Inverse difference moment | GLCM | Excellent | 0.977 (0.962, 0.987) | 5.12 | 5.68 |
| Inverse difference | GLCM | Excellent | 0.977 (0.960, 0.986) | 3.89 | 4.39 |
| Run percentage | GLRLM | Excellent | 0.977 (0.960, 0.986) | 3.93 | 3.84 |
| Run length non uniformity normalized | GLRLM | Excellent | 0.975 (0.957, 0.985) | 5.57 | 5.86 |
| Long run emphasis | GLRLM | Excellent | 0.974 (0.956, 0.985) | 10.27 | 10.04 |
| Run variance | GLRLM | Excellent | 0.973 (0.955, 0.984) | 16.86 | 18.48 |
| Difference entropy | GLCM | Excellent | 0.973 (0.954, 0.984) | 6.91 | 7.48 |
| Joint entropy | GLCM | Excellent | 0.972 (0.953, 0.984) | 8.45 | 8.91 |
| Short run emphasis | GLRLM | Excellent | 0.972 (0.953, 0.984) | 2.95 | 2.99 |
| Long run low grey level emphasis | GLRLM | Excellent | 0.968 (0.946, 0.982) | 29.62 | 31.71 |
| Large dependence emphasis | GLDM | Excellent | 0.968 (0.945, 0.981) | 13.85 | 15.52 |
| Small dependence emphasis | GLDM | Excellent | 0.966 (0.942, 0.980) | 10.53 | 11.89 |
| Dependence variance | GLDM | Excellent | 0.964 (0.939, 0.979) | 14.42 | 16.02 |
| Sum entropy | GLCM | Excellent | 0.959 (0.931, 0.976) | 7.79 | 8.21 |
| Zone percentage | GLSZM | Excellent | 0.955 (0.924, 0.974) | 13.81 | 14.96 |
| Large area low grey level emphasis | GLSZM | Excellent | 0.954 (0.922, 0.973) | 46.52 | 49.49 |
| Difference average | GLCM | Excellent | 0.952 (0.919, 0.972) | 12.64 | 14.04 |
| Dependence non uniformity normalized | GLDM | Excellent | 0.951 (0.918, 0.971) | 7.51 | 8.16 |
| Coarseness | NGTDM | Excellent | 0.949 (0.914, 0.970) | 12.45 | 13.74 |
| Gray level non uniformity normalized | GLRLM | Excellent | 0.943 (0.904, 0.967) | 12.21 | 13.33 |
| Large dependence low grey level emphasis | GLDM | Excellent | 0.943 (0.904, 0.967) | 31.97 | 34.25 |
| Low grey level run emphasis | GLRLM | Excellent | 0.936 (0.893, 0.962) | 27.8 | 29.18 |
| Low grey level emphasis | GLDM | Excellent | 0.935 (0.890, 0.962) | 28.02 | 29.34 |
| Short run low grey level emphasis | GLRLM | Excellent | 0.925 (0.874, 0.956) | 28.15 | 29.89 |
| Maximum probability | GLCM | Excellent | 0.924 (0.873, 0.955) | 20.82 | 23.01 |
| Joint energy | GLCM | Excellent | 0.917 (0.862, 0.951) | 20.95 | 23.44 |
| Dependence non uniformity | GLDM | Excellent | 0.916 (0.860, 0.950) | 12.64 | 13.89 |
| Run length non uniformity | GLRLM | Excellent | 0.913 (0.855, 0.948) | 14.98 | 15.21 |
| Zone variance | GLSZM | Excellent | 0.912 (0.853, 0.948) | 37.6 | 41.1 |
| Large area emphasis | GLSZM | Excellent | 0.911 (0.852, 0.947) | 36.81 | 40.48 |
| Gray level non uniformity | GLDM | Excellent | 0.908 (0.847, 0.946) | 15.35 | 16.58 |
| Low grey level zone emphasis | GLSZM | Excellent | 0.907 (0.846, 0.945) | 28.28 | 31.81 |
| Gray level non uniformity | GLRLM | Excellent | 0.904 (0.841, 0.943) | 13.28 | 14.21 |
| Zone entropy | GLSZM | Good | 0.899 (0.832, 0.940) | 5.45 | 5.6 |
| Sum average | GLCM | Good | 0.894 (0.824, 0.937) | 13.32 | 13.63 |
| Joint average | GLCM | Good | 0.894 (0.824, 0.937) | 13.32 | 13.63 |
| Run entropy | GLRLM | Good | 0.892 (0.821, 0.936) | 3.89 | 4.18 |
| Small dependence low grey level emphasis | GLDM | Good | 0.890 (0.818, 0.934) | 28.43 | 30.67 |
| Small area low grey level emphasis | GLSZM | Good | 0.887 (0.813, 0.933) | 30.28 | 34.2 |
| Sum of Squares | GLCM | Good | 0.886 (0.811, 0.932) | 24.96 | 28.2 |
| Cluster tendency | GLCM | Good | 0.885 (0.810, 0.931) | 27.41 | 30.75 |
| Dependence entropy | GLDM | Good | 0.884 (0.809, 0.931) | 2.65 | 2.83 |
| Small area emphasis | GLSZM | Good | 0.871 (0.788, 0.923) | 5.48 | 5.73 |
| Contrast | NGTDM | Good | 0.862 (0.775, 0.918) | 28.02 | 32.28 |
| Informal Measure of Correlation1 | GLCM | Good | 0.853 (0.760, 0.912) | 21.32 | 24 |
| Gray level non uniformity | GLSZM | Good | 0.841 (0.742, 0.905) | 17.71 | 19.56 |
| Size zone non uniformity | GLSZM | Good | 0.841 (0.742, 0.905) | 17.71 | 19.56 |
| Size zone non uniformity normalized | GLSZM | Good | 0.839 (0.739, 0.903) | 9.06 | 9.49 |
| Contrast | GLCM | Good | 0.830 (0.725, 0.898) | 22.16 | 24.62 |
| Inverse variance | GLCM | Good | 0.830 (0.725, 0.897) | 7.57 | 7.35 |
| Gray level variance | GLSZM | Good | 0.805 (0.687, 0.882) | 28.18 | 30.45 |
| Gray level variance | GLRLM | Good | 0.802 (0.682, 0.880) | 27.9 | 30.86 |
| Gray level variance | GLDM | Good | 0.801 (0.681, 0.879) | 28.06 | 31.03 |
| Large area high grey level emphasis | GLSZM | Good | 0.799 (0.677, 0.878) | 37.97 | 43.99 |
| Difference variance | GLCM | Good | 0.792 (0.667, 0.873) | 22.31 | 24.62 |
| Busyness | NGTDM | Good | 0.791 (0.665, 0.873) | 31.3 | 34.22 |
| Small dependence high grey level emphasis | GLDM | Good | 0.774 (0.641, 0.862) | 28.87 | 31.67 |
| Small area high grey level emphasis | GLSZM | Good | 0.762 (0.624, 0.855) | 25.35 | 27.64 |
| Informal measureofcorrelation2 | GLCM | Good | 0.752 (0.608, 0.848) | 13.4 | 14.27 |
| High Grey levelzone Emphasis | GLSZM | Good | 0.750 (0.606, 0.847) | 23.47 | 25.58 |
| Short run high grey level emphasis | GLRLM | Moderate | 0.737 (0.588, 0.838) | 25.14 | 26.43 |
| High grey level run emphasis | GLRLM | Moderate | 0.730 (0.577, 0.833) | 24.74 | 25.49 |
| High grey level emphasis | GLDM | Moderate | 0.728 (0.574, 0.832) | 24.87 | 25.68 |
| Autocorrelation | GLCM | Moderate | 0.722 (0.566, 0.828) | 25.4 | 26.36 |
| Correlation | GLCM | Moderate | 0.699 (0.533, 0.813) | 17.8 | 18.32 |
| Long run high grey level emphasis | GLRLM | Moderate | 0.652 (0.468, 0.782) | 26.1 | 27.79 |
| Complexity | NGTDM | Moderate | 0.589 (0.385, 0.739) | 45.63 | 51.17 |
| Large dependence high grey level emphasis | GLDM | Moderate | 0.554 (0.339, 0.714) | 26.41 | 28.55 |
| Inverse difference normalized | GLCM | Poor | 0.496 (0.267, 0.673) | 0.82 | 0.96 |
| Strength | NGTDM | Poor | 0.386 (0.136, 0.591) | 51.61 | 58.26 |
| Cluster prominence | GLCM | Poor | 0.385 (0.135, 0.590) | 61.89 | 73.39 |
| Inverse difference moment normalized | GLCM | Poor | 0.369 (0.116, 0.577) | 0.24 | 0.26 |
| Cluster shade | GLCM | Poor | -0.137 (-0.387, 0.133) | 158.52 | 87.73 |

**Supplementary Table 8 footnote:** CI: confidence interval; CV: coefficient of variation; GLCM: grey level co-occurrence matrix; GLDM: grey level dependence matrix; GLRLM: grey level run length matrix; GLSZM: grey level size zone matrix; NGTDM: neighbouring grey tone difference matrix; ICC:

intra-class correlation coefficient; LV: left ventricle; MRD: mean relative difference

**Supplementary Table 9**. **The 10 most and 10 least robust LV myocardium texture features in end-systole**

| Feature name | Robustness | ICC (95% CI) | CV (%) | MRD (%) |
| --- | --- | --- | --- | --- |
| Inverse difference moment | Excellent | 0.977 (0.962, 0.987) | 5.12 | 5.68 |
| Inverse difference | Excellent | 0.977 (0.960, 0.986) | 3.89 | 4.39 |
| Run percentage | Excellent | 0.977 (0.960, 0.986) | 3.93 | 3.84 |
| Run length non uniformity normalized | Excellent | 0.975 (0.957, 0.985) | 5.57 | 5.86 |
| Long run emphasis | Excellent | 0.974 (0.956, 0.985) | 10.27 | 10.04 |
| Run variance | Excellent | 0.973 (0.955, 0.984) | 16.86 | 18.48 |
| Difference entropy | Excellent | 0.973 (0.954, 0.984) | 6.91 | 7.48 |
| Joint entropy | Excellent | 0.972 (0.953, 0.984) | 8.45 | 8.91 |
| Short run emphasis | Excellent | 0.972 (0.953, 0.984) | 2.95 | 2.99 |
| Long run low grey level emphasis | Excellent | 0.968 (0.946, 0.982) | 29.62 | 31.71 |
| Autocorrelation | Moderate | 0.722 (0.566, 0.828) | 25.4 | 26.36 |
| Correlation | Moderate | 0.699 (0.533, 0.813) | 17.80 | 18.32 |
| Long run high grey level emphasis | Moderate | 0.652 (0.468, 0.782) | 26.10 | 27.79 |
| Complexity | Moderate | 0.589 (0.385, 0.739) | 45.63 | 51.17 |
| Large dependence high grey level emphasis | Moderate | 0.554 (0.339, 0.714) | 26.41 | 28.55 |
| Inverse difference normalized | Poor | 0.496 (0.267, 0.673) | 0.82 | 0.96 |
| Strength | Poor | 0.386 (0.136, 0.591) | 51.61 | 58.26 |
| Cluster prominence | Poor | 0.385 (0.135, 0.590) | 61.89 | 73.39 |
| Inverse difference moment normalized | Poor | 0.369 (0.116, 0.577) | 0.24 | 0.26 |
| Cluster shade | Poor | -0.137 (-0.387, 0.133) | 158.52 | 87.73 |

**Supplementary Table 9 footnote:** CI: confidence interval; CV: Coefficient of variation; ICC: intra-class correlation coefficient; MRD: Mean relative difference.
