# Supplementary material for: Repeatability of Cardiac Magnetic Resonance Radiomics: A Multi-Centre Multi-Vendor Test-Retest Study
Source: Front Cardiovasc Med. 2020 Dec 2;7:586236. doi: 10.3389/fcvm.2020.586236 (PMC7738466; doi:10.3389/fcvm.2020.586236)
Supplement: Supplementary file 2 [file Table_2.DOCX]

**Supplementary Figure 1. Mean ICC for shape radiomics across the different ROIs and cardiac phases**


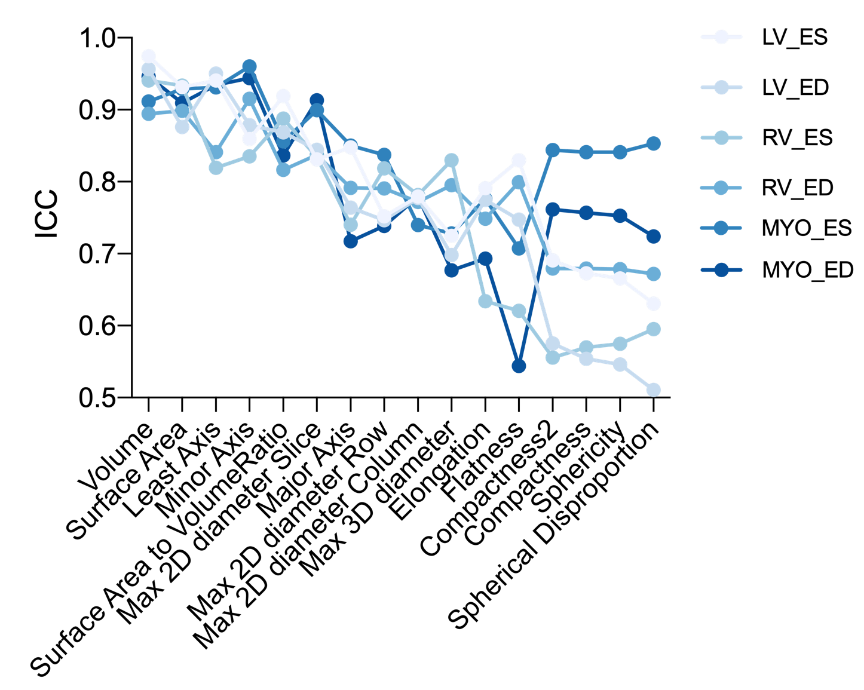


**Supplementary Figure 1 legend:** Across all three ROIs and cardiac phases, shape features quantifying volume and cavity short axis are the most reproducible and sphericity-related features are the least reproducible. ED: end-diastole; ES: end-systole; ICC: intra-class correlation; LV: left ventricle; MYO: myocardium; ROI: region of interest; RV: right ventricle.

**Supplementary Figure 2.** **Mean ICC for the different classes of texture features**


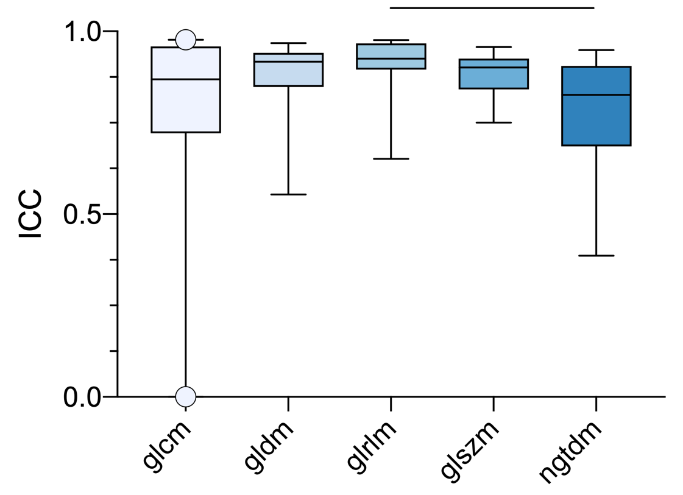


**Supplementary Figure 2 legend:** No statistically significant differences are observed between the different classes except between GLRLM and NGTDM (p = 0.0291, Dunn’s multiple comparison test). GLCM: grey level co-occurrence matrix; GLDM: grey level dependence matrix; GLRLM: grey level run length matrix; GLSZM: grey level size zone matrix; NGTDM: neighbouring grey tone difference matrix.

**Supplementary Figure 3. Mid-cavity short axis cut demonstrating minor deviation of LV endocardial contour (red) from the endocardium (yellow arrow)**


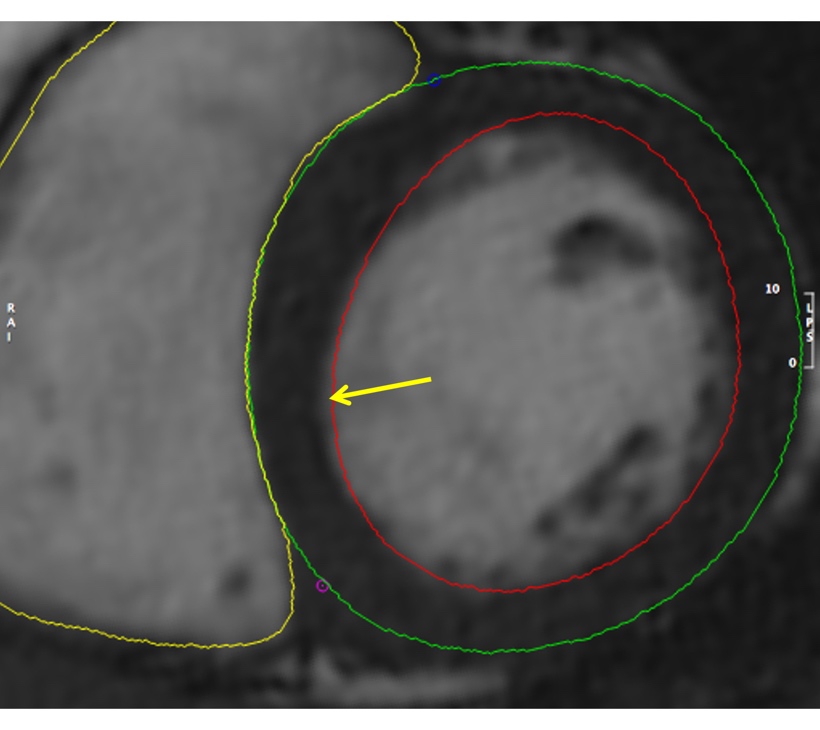


Supplementary Figure 3 footnote: The LV endocardial contour (red) is not correctly opposed to the endocardium. As a result, a small line of high intensity pixels from the LV blood pool (yellow arrow) with enter the “LV Myocardium” region of interest. These will skew the signal intensity distributions and the features which relate to this. These minor segmentation errors are not relevant for conventional CMR metrics.
